# Pharmacological Blockade of NMUR2 Suppresses Glioma Growth by Inhibiting STAT5-Mediated Transcription of Cell Cycle-associated Genes

**DOI:** 10.7150/ijbs.135596

**Published:** 2026-07-30

**Authors:** Yuna Roh, Taesang Son, Tae-Hee Han, Sarang Kim, Eunsun Jung, Jin-Seong Hwang, Joo-Young Im, Mi-Jung Kang, Mun Jeong Cho, Jung Hwa Lim, Cho-Rok Jung, Moo-Seung Lee, Hyun-Soo Cho, Dae-Soo Kim, Mi-Young Son, Seon-Young Kim, Seon-Kyu Kim, Hyun Seung Ban, Jang-Seong Kim, Tae-Su Han

**Affiliations:** 1 Korea Research Institute of Bioscience and Biotechnology (KRIBB), Daejeon 34141, Republic of Korea.; 2 University of Science and Technology (UST), Daejeon 34141, Republic of Korea.; 3 School of Medicine, Sungkyunkwan University, Suwon 16419, Republic of Korea.

**Keywords:** G-protein-coupled receptors, neuromedin U receptor 2, glioma, NNC 05-2090, temozolomide

## Abstract

Glioma is a highly aggressive brain tumor with poor prognosis and limited therapeutic options. Although temozolomide (TMZ) remains the standard chemotherapeutic agent for glioma, frequent recurrence and the development of therapeutic resistance continue to limit clinical benefit, highlighting the need for new molecular targets and treatment strategies. Here, we identify neuromedin U receptor 2 (NMUR2) as a driver of glioma progression and a potential therapeutic target. NMUR2 expression was markedly elevated in glioma tissues and positively associated with tumor grade. Functional analyses showed that NMUR2 promoted glioma cell proliferation and migration, whereas NMUR2 silencing attenuated these malignant phenotypes. Mechanistically, NMUR2 activated Gαq-dependent Ca²⁺ signaling, leading to STAT5 phosphorylation and subsequent transcriptional upregulation of the cell cycle-associated genes PIM1 and FOXM1. Drug-repurposing screening of 6,331 compounds identified NNC 05-2090 as a candidate NMUR2 antagonist. NNC 05-2090 blocked NMUR2-mediated Gαq/Ca²⁺/STAT5 signaling, which was associated with reduced PIM1 and FOXM1 expression, cell cycle arrest, and suppression of glioma growth *in vitro* and *in vivo*. In addition, combination treatment with TMZ produced synergistic anti-tumor effects in glioma models. Collectively, our findings define a previously unrecognized NMUR2/Gαq/STAT5/PIM1-FOXM1 signaling axis in glioma and support pharmacological inhibition of NMUR2 as a potential therapeutic strategy.

## Introduction

Gliomas account for approximately 30% of all brain and central nervous system (CNS) tumors and nearly 80% of malignant brain tumors, yet their pathogenic mechanisms and therapeutic vulnerabilities remain incompletely defined 1. Epidemiological data underscore their impact; the Central Brain Tumor Registry of the United States (CBTRUS) reports an overall age-standardized incidence of glial tumors of 5.95 per 100,000, with glioblastoma representing the most common malignant subtype (3.23 per 100,000) and exhibiting the poorest survival outcomes 2, 3. Despite advances in molecular classification and multidisciplinary management, effective treatment options remain limited and long-term survival is poor. Temozolomide (TMZ), an oral alkylating agent that crosses the blood-brain barrier, remains a mainstay of therapy for newly diagnosed gliomas 4. However, the median survival of patients with malignant gliomas remains less than 15 months, and approximately 55% of patients who initially respond to TMZ eventually develop resistance, resulting in recurrence and treatment failure 5, 6. These challenges highlight the need for the identification of novel molecular targets to improve therapeutic outcomes in glioma.

G protein-coupled receptors (GPCRs) are a large family of seven-transmembrane receptors that regulate diverse intracellular signaling pathways through coupling to multiple G proteins, including Gs, Gi/o, Gq/11, and G12/13 7. The human genome encodes over 800 GPCRs, which control a broad range of physiological processes 8, 9. Because of their cell-surface localization and pharmacological tractability, GPCRs are among the most intensively pursued target classes in drug discovery 10. Indeed, approximately 35% of FDA-approved drugs act on GPCRs. GPCR dysregulation has been implicated in diverse diseases, including CNS disorders, cardiovascular and metabolic diseases, inflammation, pain, and cancer 7, 11. In oncology, GPCR signaling can promote key malignant phenotypes such as proliferation, invasion, migration, metastasis, and survival 12-15. Several GPCR-targeting agents are already used in oncology or cancer-related clinical settings; for example, plerixafor, a CXCR4 antagonist, is approved in combination with G-CSF to mobilize hematopoietic stem cells for autologous transplantation in patients with non-Hodgkin's lymphoma and multiple myeloma 16. Degarelix and octreotide are also used in prostate cancer and neuroendocrine tumors, respectively. However, GPCR-directed therapeutic strategies remain relatively underexplored in glioma, highlighting the need to identify GPCRs that contribute to glioma progression and may serve as therapeutic targets.

Neuromedin U receptor 2 (NMUR2) is a GPCR that primarily binds neuromedin U (NMU), a neuropeptide involved in diverse physiological processes. NMUR2 shares 50% amino-acid sequence identity with NMUR1 17, 18, yet the two receptors exhibit distinct tissue distributions and signaling functions. NMUR1 is predominantly expressed in peripheral tissues and is linked to immune and metabolic regulation, whereas NMUR2 is most abundantly expressed in the CNS and regulates behaviors related to feeding, reward, and neuroendocrine function 19-21. Aberrant NMUR2 expression has been reported in several malignancies, including breast, colorectal, endometrial, and ovarian cancers 22-25, suggesting a potential role in tumor progression. Consistent with this notion, NMUR2 has been associated with oncogenic signaling through pathways such as MAPK and PI3K/Akt, as well as with pro-migratory and pro-invasive phenotypes, potentially involving angiogenic and epithelial-mesenchymal transition-related programs. Given its predominant expression in the CNS and emerging roles in cancer, NMUR2 represents an attractive but insufficiently explored candidate regulator of glioma biology.

In the present study, we investigated the role of NMUR2 in human glioma and evaluated its therapeutic potential. We show that NMUR2 promotes glioma cell growth through activation of Gαq/Ca²⁺-dependent STAT5 signaling, leading to transcriptional upregulation of cell cycle-associated genes. We further demonstrate that pharmacological inhibition of NMUR2 suppresses this signaling axis and represents a potential therapeutic strategy for glioma.

## Results

### NMUR2 is upregulated in glioma and promotes oncogenic phenotypes

**Figure 1:**
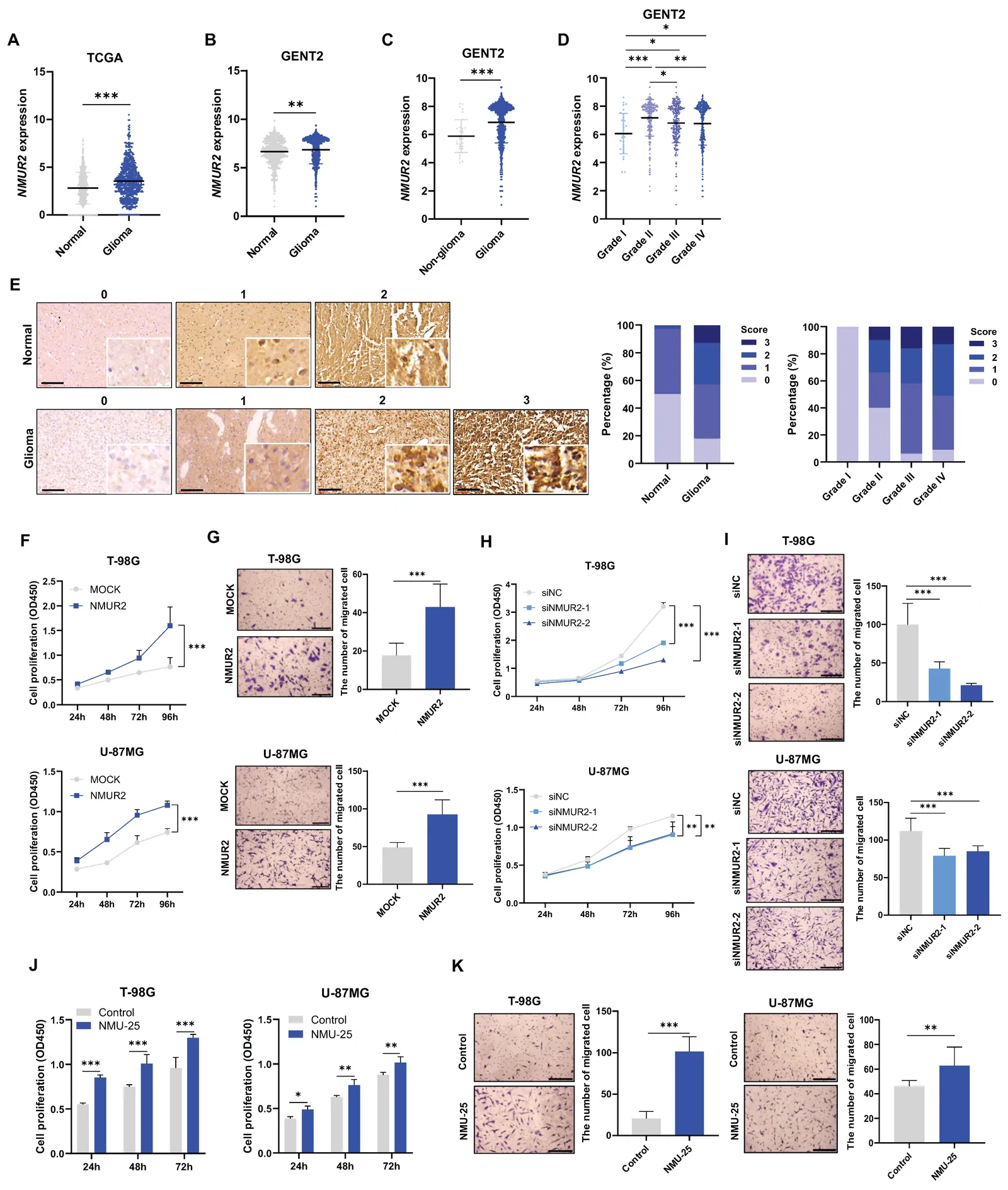
** NMUR2 is upregulated in glioma and promotes oncogenic phenotypes.** A. *NMUR2* expression in human normal brain tissues (n = 1141) and glioma tissues (LGG + GBM, n = 689) based on TCGA dataset analysis. B-D. NMUR2 expression analysis using the GENT2 database. B. Comparison of *NMUR2* expression between normal brain tissues (n = 873) and glioma tissues (n = 946). C. Comparison of *NMUR2* expression between non-glioma brain tumor samples (n = 32) and glioma tissues (n = 946). D. *NMUR2* expression according to glioma grade. E. Representative immunohistochemical staining of NMUR2 in normal brain and glioma tissues using a tissue microarray. NMUR2 expression levels are shown according to glioma grade. Scale bar, 100 μm. F. Cell proliferation in T-98G and U-87MG cells transfected with mock or NMUR2 expression vector. Cell proliferation was assessed by CCK-8 assay. G. Cell migration in T-98G and U-87MG cells transfected with mock or NMUR2 expression vector. Cell migration was evaluated using a Transwell migration assay, and migrated cells were visualized by crystal violet staining. Scale bar, 500 μm. H. Cell proliferation in T-98G and U-87MG cells transfected with non-targeting control siRNA (siNC) or NMUR2-targeting siRNA (siNMUR2). Cell proliferation was assessed by CCK-8 assay. I. Cell migration in T-98G and U-87MG cells transfected with siNC or siNMUR2. Cell migration was analyzed using a Transwell migration assay. J. Cell proliferation in T-98G and U-87MG cells treated with vehicle or NMU-25. K. Cell migration in T-98G and U-87MG cells treated with vehicle or NMU-25. Data are presented as mean ± SD. *P < 0.05, **P < 0.01, ***P < 0.001.

To determine whether NMUR2 is dysregulated in glioma, we first analyzed its expression in publicly available datasets. Analysis of TCGA data showed that NMUR2 expression was significantly higher in lower-grade glioma (LGG) and glioblastoma (GBM) tissues than in normal brain tissues (Fig. 1A). Consistent with this finding, microarray data from the GENT2 database also revealed significantly increased NMUR2 expression in glioma samples compared with normal controls (Fig. 1B). In addition, NMUR2 expression was markedly higher in glioma samples than in non-glioma brain tumor samples (Fig. 1C). Stratification by tumor grade further showed that NMUR2 expression was significantly elevated in grade II-IV gliomas compared with grade I gliomas (Fig. 1D). We also examined the expression of NMU, the endogenous ligand of NMUR2, in the same datasets. Although NMU expression was significantly increased in glioma samples in the TCGA dataset (Fig. S1A), its expression pattern was less consistent in the GENT2 dataset, showing no clear difference between normal brain and glioma tissues and lower expression in glioma than in non-glioma brain tumor samples (Fig. S1B-C). Notably, however, NMU expression increased with glioma grade (Fig. S1D). Because NMUR2 showed a more consistent pattern of upregulation across datasets and grades, we focused subsequent analyses on NMUR2 as the principal component of this signaling axis in glioma.

We further extended this investigation to clinical samples, in which NMUR2 expression was examined using a tissue array of glioma and normal brain tissues. Our findings revealed an upregulation of NMUR2 expression in glioma tissues relative to that in normal brain tissues (Fig. 1E). Notably, NMUR2 expression levels were positively correlated with glioma grade, indicating its potential role in tumor progression.

We next examined whether NMUR2 affects malignant phenotypes in glioma cells. Ectopic overexpression of NMUR2 in T-98G and U-87MG cells (Fig. S1E-F) significantly increased cell proliferation and migration (Fig. 1F-G). Conversely, knockdown of NMUR2 in T-98G and U-87MG cells (Fig. S1G-H) significantly reduced both cell proliferation and migration (Fig. 1H-I). In addition, treatment of GBM cell lines with NMU-25, an agonist of NMUR2, significantly enhanced cell proliferation and migration (Fig. 1J-K). Together, these results demonstrate that NMUR2 is upregulated in glioma and promotes proliferative and migratory phenotypes, suggesting a role for NMUR2 in glioma progression.

### NMUR2 promotes glioma cell proliferation through regulation of cell cycle-associated genes

**Figure 2:**
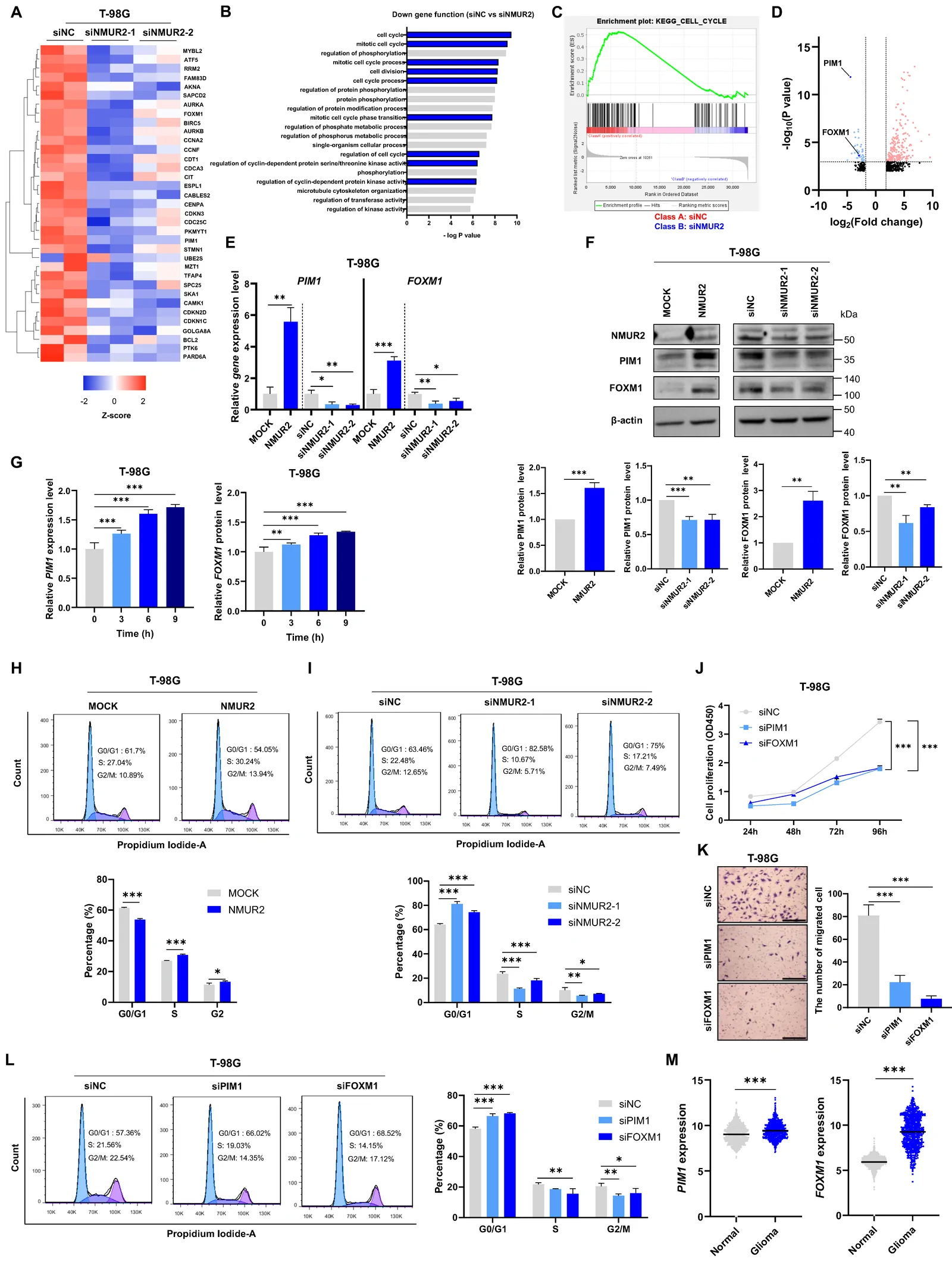
** NMUR2 expression and activation regulate cell cycle-associated genes and cell cycle progression.** A. Heatmap of RNA-sequencing data from T-98G cells transfected with siNC or siNMUR2. B. Gene ontology analysis of downregulated biological functions in T-98G cells transfected with siNMUR2. C. Gene Set Enrichment Analysis (GSEA) plot showing enrichment of the KEGG cell cycle pathway in T-98G cells transfected with siNC or siNMUR2. D. Volcano plot of differentially expressed genes in T-98G cells transfected with siNC or siNMUR2. Blue and red dots indicate genes with lower and higher expression levels in siNMUR2-transfected cells, respectively. The y-axis indicates -log10 (*P* value), and the x-axis indicates log2 (fold change). E-F. *PIM1* and *FOXM1* expression in T-98G cells following NMUR2 overexpression or knockdown. E. mRNA expression determined by real-time PCR. F. Protein expression determined by western blotting. G. Time-course analysis of *PIM1* and *FOXM1* mRNA expression in T-98G cells treated with NMU-25. mRNA levels were measured at 3-h intervals by real-time PCR. H-I. Cell cycle analysis of T-98G cells transfected with NMUR2 expression vector or NMUR2-targeting siRNA. H. Cells transfected with empty vector or NMUR2 expression vector. I. Cells transfected with siNC, siNMUR2-1, or siNMUR2-2. After 48 h of transfection, cells were stained with propidium iodide and analyzed by flow cytometry to determine DNA content in G1, S, and G2/M phases. Graphs show cell cycle distribution. J. Cell proliferation in T-98G cells transfected with siNC, siPIM1, or siFOXM1. Cell proliferation was assessed by CCK-8 assay. K. Cell migration in T-98G cells transfected with siNC, siPIM1, or siFOXM1. Cell migration was evaluated using a Transwell migration assay, and migrated cells were visualized by crystal violet staining. Scale bar, 500 μm. L. Cell cycle analysis of T-98G cells transfected with siNC, siPIM1, or siFOXM1. After 48 h of transfection, cells were stained with propidium iodide and analyzed by flow cytometry to determine DNA content in G1, S, and G2/M phases. Graph shows cell cycle distribution. M. *PIM1* (left) and *FOXM1* (right) expression in human normal brain tissues (n = 1141) and glioma tissues (LGG + GBM, n = 689) based on TCGA dataset analysis. Data are presented as means ± SD (*P < 0.05, **P < 0.01, ***P < 0.001).

To investigate the oncogenic function of NMUR2, we performed RNA sequencing in T-98G cells transfected with control siRNA (siNC) or NMUR2-targeting siRNA (siNMUR2). NMUR2 knockdown caused marked changes in global gene expression profiles compared with the control group (Fig. 2A). Gene Ontology (GO) analysis and Gene Set Enrichment Analysis (GSEA) showed that genes associated with cell cycle regulation were significantly enriched among the differentially expressed genes affected by NMUR2 depletion (Fig. 2B-C). Among these, PIM1 and FOXM1, which are key regulators of cell cycle progression, were identified as potential downstream effectors of NMUR2 signaling (Fig. 2D). We next examined whether NMUR2 regulates the expression of these genes. Overexpression of NMUR2 in T-98G cells increased both the mRNA and protein levels of PIM1 and FOXM1, whereas knockdown of NMUR2 markedly reduced their expression (Fig. 2E-F). In addition, stimulation with NMU-25, an agonist of NMUR2, significantly increased the mRNA levels of *PIM1* and *FOXM1* (Fig. 2G).

To further define the role of NMUR2 in cell cycle regulation, we performed flow cytometric analysis in NMUR2-overexpressing and NMUR2-depleted cells. NMUR2 overexpression increased the proportion of cells in S phase, whereas NMUR2 knockdown induced G0/G1 arrest (Fig. 2H-I). Consistent with these findings, NMUR2 overexpression increased the expression of cell cycle-promoting genes, including *CyclinB1* and *CyclinD1*, whereas NMUR2 depletion reduced their expression. In contrast, *CDKN1B* (p27), a negative regulator of cell cycle progression, showed an opposite expression pattern (Fig. S2A).

To determine whether PIM1 and FOXM1 contribute to NMUR2-mediated glioma progression, we silenced each gene using siRNA. Knockdown of PIM1 or FOXM1 significantly reduced cell proliferation and migration in T-98G cells (Fig. 2J-K and Fig. S2B). In addition, silencing of these genes increased cell cycle arrest, further supporting their role in NMUR2-driven cell cycle progression (Fig. 2L). Consistent with their proposed relevance in glioma, analysis of the TCGA and GENT2 datasets showed that PIM1 and FOXM1 expression levels were significantly higher in glioma tissues than in normal brain tissues (Fig. 2M and Fig. S2C). Together, these findings indicate that NMUR2 promotes glioma cell proliferation by regulating cell cycle-associated genes, including *PIM1* and *FOXM1*.

### NMUR2 regulates transcription of cell cycle-associated genes through STAT5 binding to their promoters

**Figure 3:**
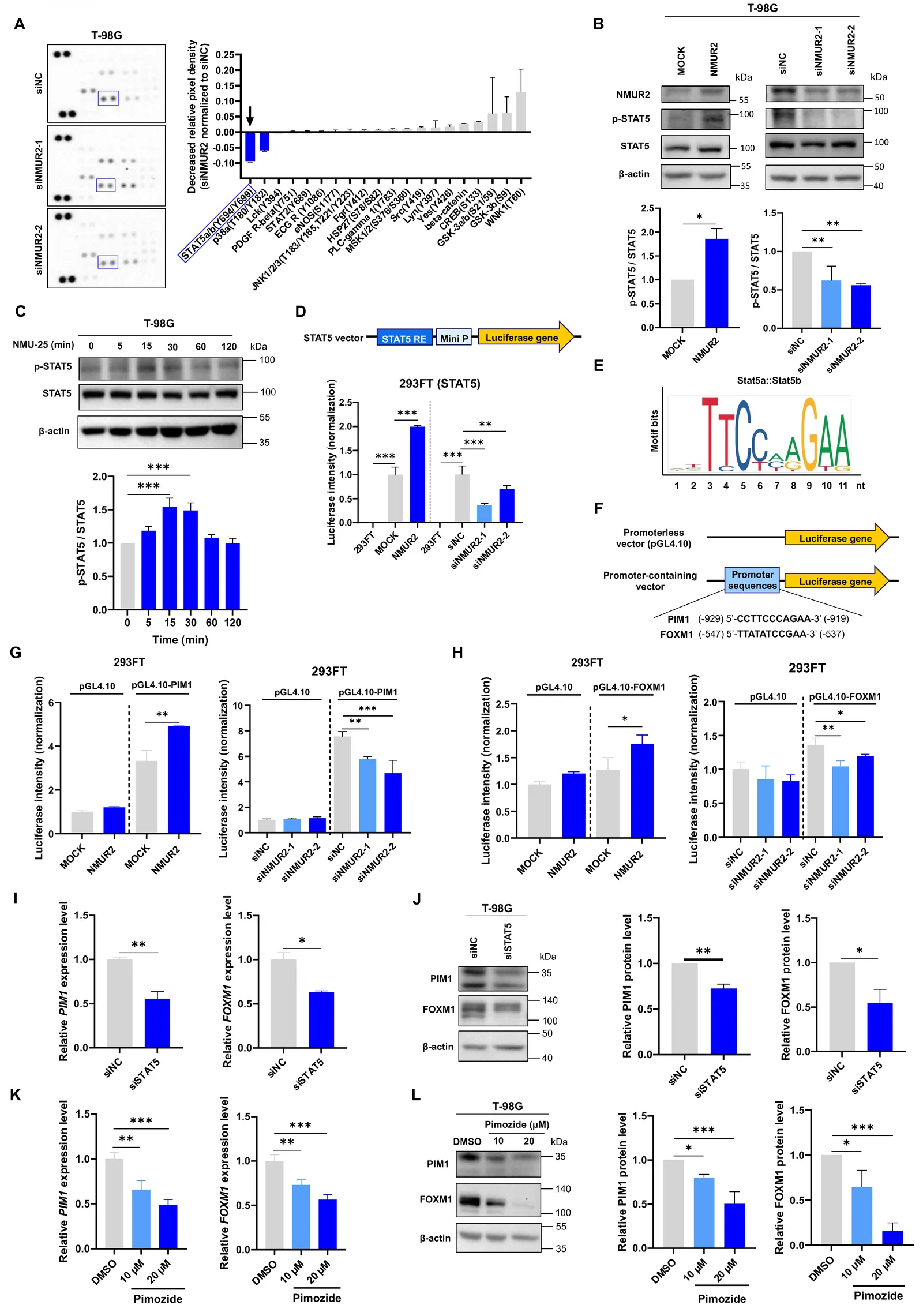
** STAT5 mediates NMUR2-dependent regulation of PIM1 and FOXM1 transcription.** A. Phosphokinase array analysis of T-98G cells transfected with siNC or siNMUR2 for 48 h. STAT5a/b (Y694/Y699) is indicated. B-C. STAT5 phosphorylation in T-98G cells. B. Phospho-STAT5 and total STAT5 levels in T-98G cells following NMUR2 overexpression or knockdown, determined by western blotting. C. Time-dependent phosphorylation of STAT5 in T-98G cells following NMU-25 treatment (0, 5, 15, 30, 60, and 120 min), determined by western blotting. Phospho-STAT5 levels were quantified relative to total STAT5. D. Luciferase reporter assay in cells transfected with a reporter vector containing STAT5 response elements following NMUR2 overexpression or knockdown. E. Predicted STAT5A:STAT5B binding motif identified using the JASPAR database (https://jaspar.elixir.no/). F. Schematic diagram of the PIM1 and FOXM1 promoter regions containing predicted STAT5-binding sites. Promoter regions were identified using the Eukaryotic Promoter Database (EPD, https://epd.expasy.org/epd/) and cloned into the promoterless pGL4.10 vector. G-H. Luciferase reporter assay using empty vector, pGL4.10-PIM1 (G), or pGL4.10-FOXM1 (H) following NMUR2 overexpression or knockdown. I-J. PIM1 and FOXM1 expression in T-98G cells following siRNA-mediated STAT5 knockdown. I. *PIM1* and *FOXM1* mRNA expression determined by RT-qPCR. J. PIM1 and FOXM1 protein expression determined by western blotting. K-L. PIM1 and FOXM1 expression in T-98G cells treated with the indicated concentrations of Pimozide. K. *PIM1* and *FOXM1* mRNA expression determined by RT-qPCR. L. PIM1 and FOXM1 protein expression determined by western blotting. Data are presented as means ± SD (*P < 0.05, **P < 0.01, ***P < 0.001).

To investigate how NMUR2 regulates downstream signaling, we focused on kinase phosphorylation, a common mechanism in GPCR-mediated pathways. A phosphokinase array was therefore performed in T-98G cells transfected with siNC or siNMUR2 to identify signaling molecules that may regulate the expression of cell cycle-associated genes, including PIM1 and FOXM1. Notably, the array revealed a reduction in phospho-STAT5 levels upon NMUR2 knockdown (Fig. 3A). This result was further validated by western blot analysis, which demonstrated that STAT5 phosphorylation increases with NMUR2 overexpression and decreases upon NMUR2 knockdown (Fig. 3B and Fig. S3A). Additionally, NMU-25 stimulation induced a time-dependent increase in STAT5 phosphorylation, with prominent phosphorylation observed at 15-30 min after treatment in glioma cells (Fig. 3C and Fig. S3B). These findings further support that NMUR2 activation promotes STAT5 signaling, potentially contributing to the transcriptional regulation of cell cycle-associated genes such as *PIM1* and *FOXM1*.

Because STAT5 functions as a transcriptional activator, we next examined whether NMUR2 affects STAT5-dependent transcriptional activity. A luciferase reporter assay using a vector containing a STAT5 response element showed that reporter activity was increased by NMUR2 overexpression and reduced by NMUR2 knockdown (Fig. 3D), indicating that NMUR2 enhances STAT5-dependent transcription. We then investigated whether STAT5 directly regulates PIM1 and FOXM1 transcription. Putative STAT5-binding motifs within the promoter regions of PIM1 and FOXM1 were identified using the JASPAR database, and their promoter locations were further annotated using the Eukaryotic Promoter Database (EPD) (Fig. 3E). Based on these analyses, luciferase reporter constructs containing the PIM1 or FOXM1 promoter regions were generated (Fig. 3F). In promoter activity assays, NMUR2 overexpression significantly increased PIM1 promoter-driven luciferase activity, whereas NMUR2 knockdown reduced it (Fig. 3G). A similar pattern was observed for the FOXM1 promoter, with increased activity upon NMUR2 overexpression and reduced activity following NMUR2 knockdown (Fig. 3H).

To further determine whether STAT5 contributes to the regulation of PIM1 and FOXM1 expression, we examined the effects of siRNA-mediated and pharmacological suppression of STAT5. Efficient STAT5 knockdown was confirmed in glioma cells (Fig. S3C). STAT5 depletion reduced the mRNA levels of *PIM1* and *FOXM1* (Fig. 3I and Fig. S3D) and also reduced their protein expression (Fig. 3J and Fig. S3E). Consistently, treatment with Pimozide, a pharmacological STAT5 inhibitor, decreased *PIM1* and *FOXM1* mRNA expression in a concentration-dependent manner (Fig. 3K and Fig. S3F), as well as the protein levels of both PIM1 and FOXM1 (Fig. 3L and Fig. S3G). Together with the promoter reporter assays, these findings indicate that NMUR2 promotes PIM1 and FOXM1 expression through STAT5-dependent transcriptional regulation, supporting the role of the NMUR2-STAT5 axis in regulating cell cycle-associated gene expression.

### NMUR2 regulates the expression of cell cycle-associated genes through the Gαq protein-STAT5 pathway

**Figure 4:**
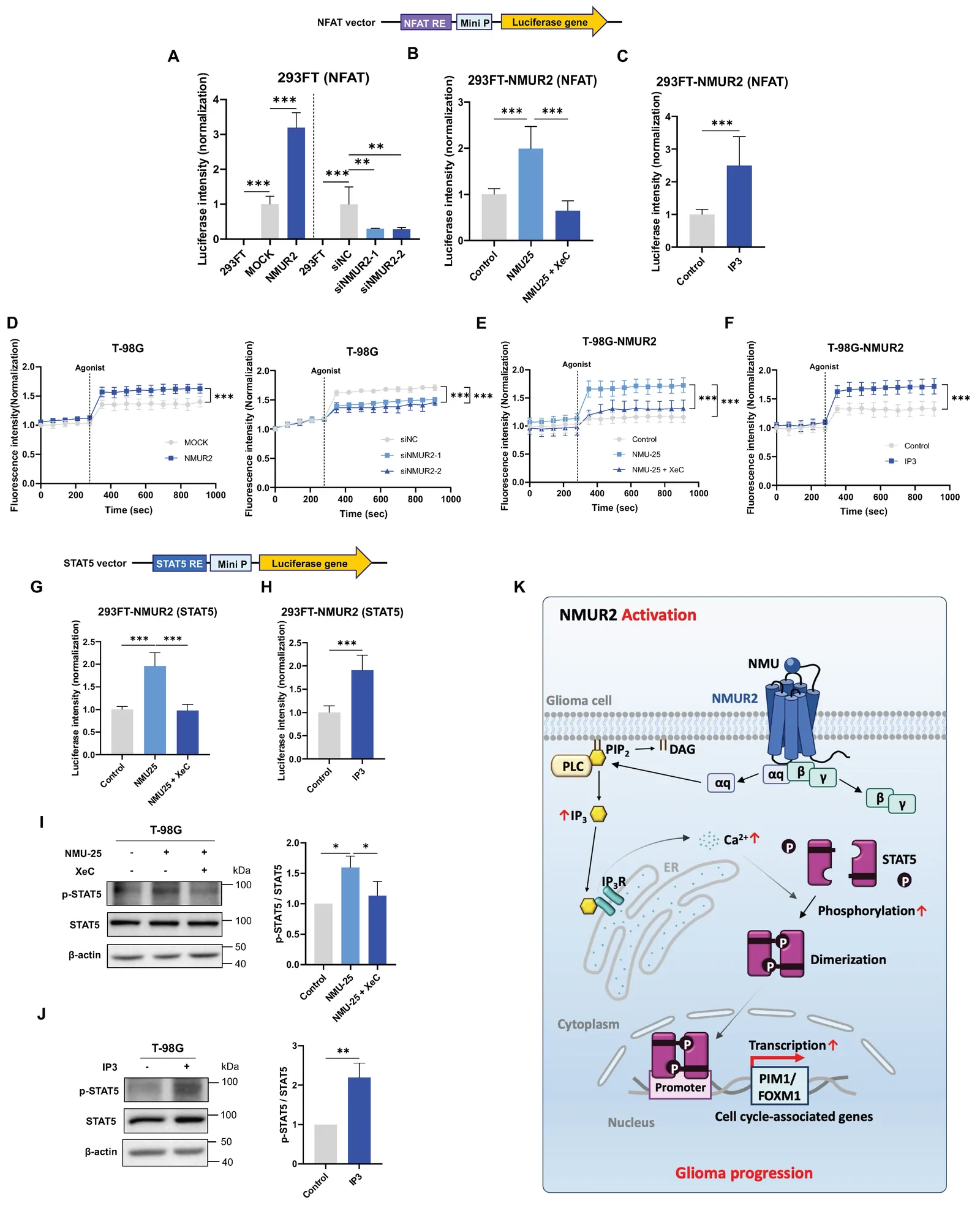
** NMUR2-induced Gαq-mediated Ca²⁺ mobilization regulates STAT5 phosphorylation.** A-C. Luciferase reporter assay using a construct containing Nuclear Factor of Activated T-cells (NFAT) response elements in 293FT cells. A. Luciferase activity in 293FT cells following NMUR2 overexpression or knockdown. B. Luciferase activity in NMUR2-overexpressing 293FT cells treated with NMU-25 and Xestospongin C (XeC), an IP3 receptor inhibitor. C. Luciferase activity in NMUR2-overexpressing 293FT cells treated with IP3. D-F. Intracellular Ca²⁺ analysis in T-98G cells using Green FLUOFORTE Dye. D. Intracellular Ca²⁺ levels in T-98G cells following NMUR2 overexpression or knockdown. E. Intracellular Ca²⁺ levels in T-98G cells treated with NMU-25 and XeC. F. Intracellular Ca²⁺ levels in T-98G cells treated with IP3. G-H. Luciferase reporter assay using a construct containing STAT5 response elements in NMUR2-overexpressing 293FT cells. G. Luciferase activity in cells treated with NMU-25 and XeC. H. Luciferase activity in cells treated with IP3. I-J. STAT5 phosphorylation in T-98G cells determined by western blotting. I. Phospho-STAT5 and total STAT5 levels in cells treated with NMU-25 and XeC. J. Phospho-STAT5 and total STAT5 levels in cells treated with IP3. K. Schematic illustration of the proposed NMUR2 signaling pathway involving Gαq-mediated Ca²⁺ mobilization, STAT5 phosphorylation, and regulation of PIM1 and FOXM1. Data are presented as means ± SD (*P < 0.05, **P < 0.01, ***P < 0.001).

Previous studies have shown that NMUR2 signaling induces intracellular Ca²⁺ release from the endoplasmic reticulum (ER) through activation of Gαq 21. To determine whether this signaling pathway operates in glioma cells, we performed a luciferase reporter assay using a nuclear factor of activated T cells (NFAT) reporter. NFAT is activated downstream of intracellular Ca²⁺ mobilization through the Ca²⁺/calcineurin pathway; therefore, NFAT-dependent luciferase activity can be used as a surrogate readout of Gαq-mediated Ca²⁺ signaling 26, 27. In 293FT cells, luciferase activity increased with elevated NMUR2 expression and decreased with reduced NMUR2 expression (Fig. 4A). In addition, NMU-25 further enhanced NFAT reporter activity in NMUR2-overexpressing cells, whereas the IP3 receptor inhibitor Xestospongin C significantly reduced this effect (Fig. 4B). Direct treatment with IP3 also increased reporter activity in NMUR2-overexpressing 293FT cells (Fig. 4C). These findings suggest that NMUR2 activates Gαq-dependent Ca²⁺ signaling.

To further assess Ca²⁺ mobilization downstream of NMUR2, we performed a FLUOFORTE calcium assay. Intracellular Ca²⁺ levels were significantly increased in NMUR2-overexpressing T-98G cells, whereas they were reduced in NMUR2-depleted T-98G and 293FT cells (Fig. 4D and Fig. S4A). Moreover, treatment with NMU-25 or IP3 enhanced Ca²⁺ mobilization in NMUR2-overexpressing T-98G and 293FT cells, whereas Xestospongin C suppressed this response (Fig. 4E-F and Fig. S4B-C). Together, these results indicate that NMUR2 promotes intracellular Ca²⁺ release through the Gαq/IP3 signaling pathway.

We next investigated whether NMUR2-dependent Ca²⁺ signaling contributes to STAT5 activation. A STAT5-responsive luciferase reporter assay showed that NMU-25 significantly increased STAT5-dependent transcriptional activity, whereas Xestospongin C reversed this effect (Fig. 4G). In addition, treatment with IP3 increased STAT5 reporter activity in NMUR2-overexpressing 293FT cells (Fig. 4H). Consistent with these findings, western blot analysis showed that NMU-25 or IP3 treatment increased STAT5 phosphorylation, whereas inhibition of the IP3 receptor reduced phospho-STAT5 levels in T-98G cells (Fig. 4I-J). Collectively, these findings indicate that NMUR2 activates the Gαq/Ca²⁺ signaling pathway, which in turn promotes STAT5 phosphorylation and contributes to the transcriptional regulation of cell cycle-associated genes such as *PIM1* and *FOXM1* (Fig. 4K).

### Identification of NNC 05-2090 as a candidate NMUR2 antagonist

**Figure 5:**
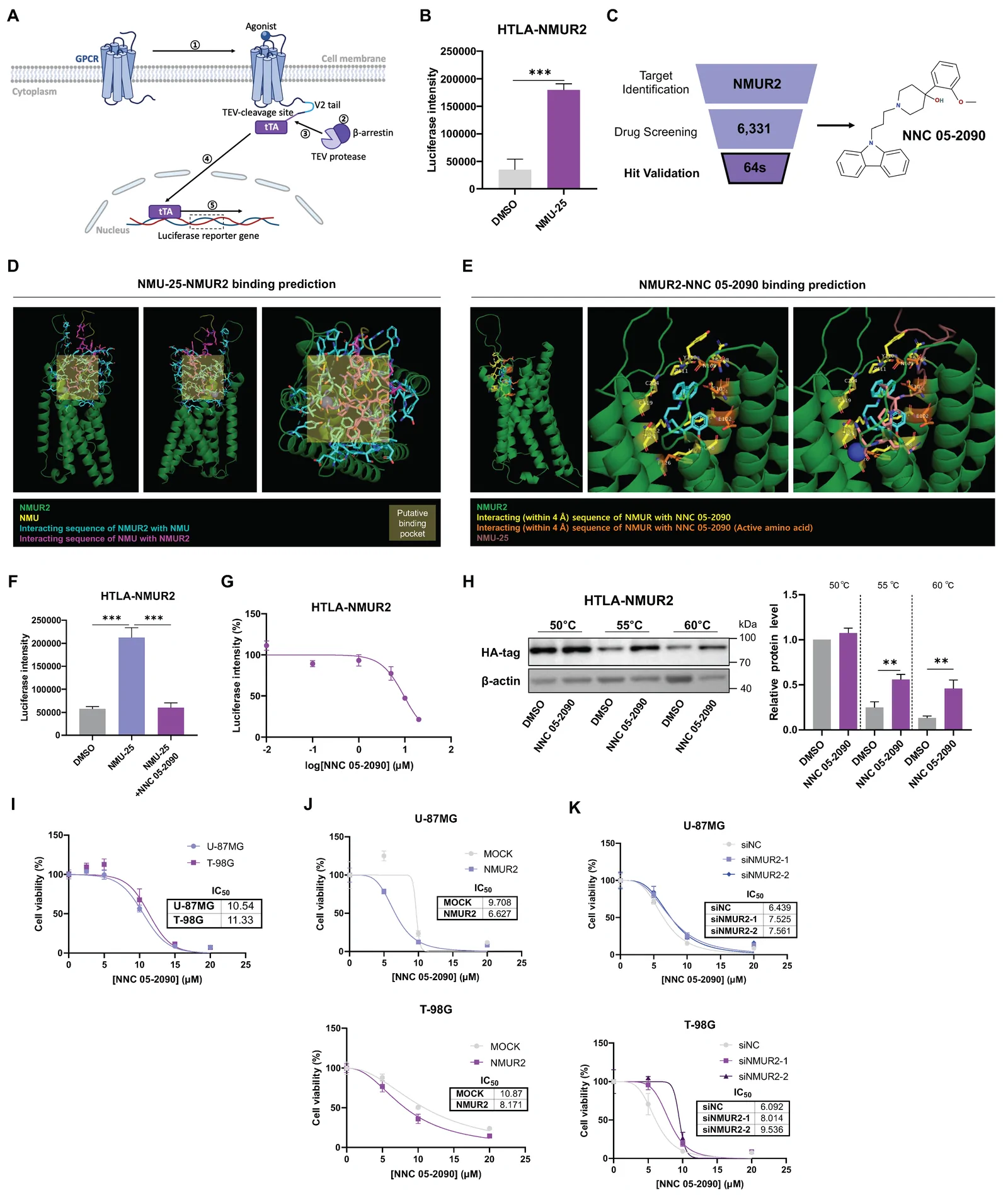
** NNC 05-2090 identified from the NMUR2-specific drug screening system interacts with NMUR2.** A. Schematic representation of the NMUR2-specific PRESTO-TANGO assay used for drug screening. Binding of NMU-25 to NMUR2 activates the luciferase reporter system through the β-arrestin/TEV protease pathway. B. Luciferase activity in NMUR2-HTLA cells treated with DMSO or NMU-25 to evaluate β-arrestin activity. C. Schematic overview of the selection process for NNC 05-2090 from the drug repurposing library containing 6,331 compounds. D-E. Molecular docking analysis of ligand binding to NMUR2. D. Predicted binding mode of NMU-25 to NMUR2. E. Predicted binding mode of NNC 05-2090 to NMUR2. Structural modeling was performed using Pharmaco-Net operated by CALICI. F. Luciferase activity in NMUR2-HTLA cells treated with NMU-25 or co-treated with NMU-25 and NNC 05-2090. G. Luciferase activity in NMUR2-HTLA cells treated with the indicated concentrations of NNC 05-2090. H. Cellular Thermal Shift Assay (CETSA) analysis of NMUR2 in cells treated with NNC 05-2090. Protein levels are quantified in the adjacent graph. I. Dose-response curves of NNC 05-2090 in U-87MG and T-98G cells for IC_50_ determination. J. Dose-response curves of NNC 05-2090 in U-87MG and T-98G cells transfected with NMUR2 expression vector for IC_50_ determination. K. Dose-response curves of NNC 05-2090 in U-87MG and T-98G cells transfected with NMUR2-targeting siRNA for IC_50_ determination. Data are presented as means ± SD (*P < 0.05, **P < 0.01, ***P < 0.001).

Based on our finding that NMUR2 drives a pro-tumorigenic signaling pathway in glioma, we next investigated whether pharmacological inhibition of NMUR2 could represent a potential therapeutic strategy. To identify compounds that antagonize NMUR2, we performed a drug-repurposing screen using the PRESTO-Tango assay system, as previously described 28. In this assay, receptor activation induces β-arrestin recruitment, which leads to TEV protease-mediated release of the tTA transcription factor and subsequent induction of luciferase expression (Fig. 5A). To establish the screening platform, HTLA cells were transfected with the NMUR2-Tango vector to generate a stable NMUR2-HTLA cell line (Fig. S5A). Treatment with NMU-25 markedly increased luciferase activity in NMUR2-HTLA cells compared with untreated controls, confirming the successful establishment of an NMUR2-responsive screening system (Fig. 5B).

Using this platform, we screened a 6,331-compound drug-repurposing library and identified 64 initial hit compounds on the basis of reduced luciferase activity. Among these candidates, NNC 05-2090, a compound previously reported to inhibit the GABA transporter BGT-1 and to exhibit anticonvulsant properties 29, 30, was selected for further analysis because it showed no apparent cytotoxicity during screening and produced a sustained inhibitory effect on NMUR2 activity (Fig. 5C). In addition, comparative analysis across multiple GPCR screening datasets showed that NNC 05-2090 exhibited the strongest inhibitory effect toward NMUR2 among the receptors examined (Fig. S5B).

To further investigate the interaction between NNC 05-2090 and NMUR2, we performed *in silico* binding analysis based on previously reported structural information for NMUR2 31. Computational modeling identified a putative ligand-binding pocket within NMUR2 (Fig. 5D) and predicted that NNC 05-2090 occupies a binding region similar to that of NMU-25 (Fig. 5E and Fig. S5C), supporting the possibility that NNC 05-2090 directly interacts with NMUR2. Consistent with this prediction, treatment of NMUR2-HTLA cells with NNC 05-2090 significantly attenuated NMU-25-induced luciferase activity in a dose-dependent manner (Fig. 5F-G). Moreover, cellular thermal shift assay (CETSA) showed increased NMUR2 protein stability in cells treated with NNC 05-2090 (Fig. 5H), further supporting direct target engagement.

We next evaluated the anti-glioma activity of NNC 05-2090 in GBM cell lines. The IC_50_ values of NNC 05-2090 ranged from 10.54 to 11.33 μM, depending on the cell line examined (Fig. 5I). To determine whether NMUR2 expression influences drug responsiveness, GBM cells with altered NMUR2 expression were treated with NNC 05-2090. NMUR2 overexpression increased sensitivity to NNC 05-2090, as reflected by lower IC_50_ values (Fig. 5J). In contrast, NMUR2 knockdown reduced sensitivity to NNC 05-2090 and resulted in increased IC_50_ values (Fig. 5K). Together, these findings identify NNC 05-2090 as a candidate NMUR2 antagonist and support its selective inhibitory activity in NMUR2-dependent glioma cells.

### NNC 05-2090 inhibits NMUR2 downstream signaling

**Figure 6:**
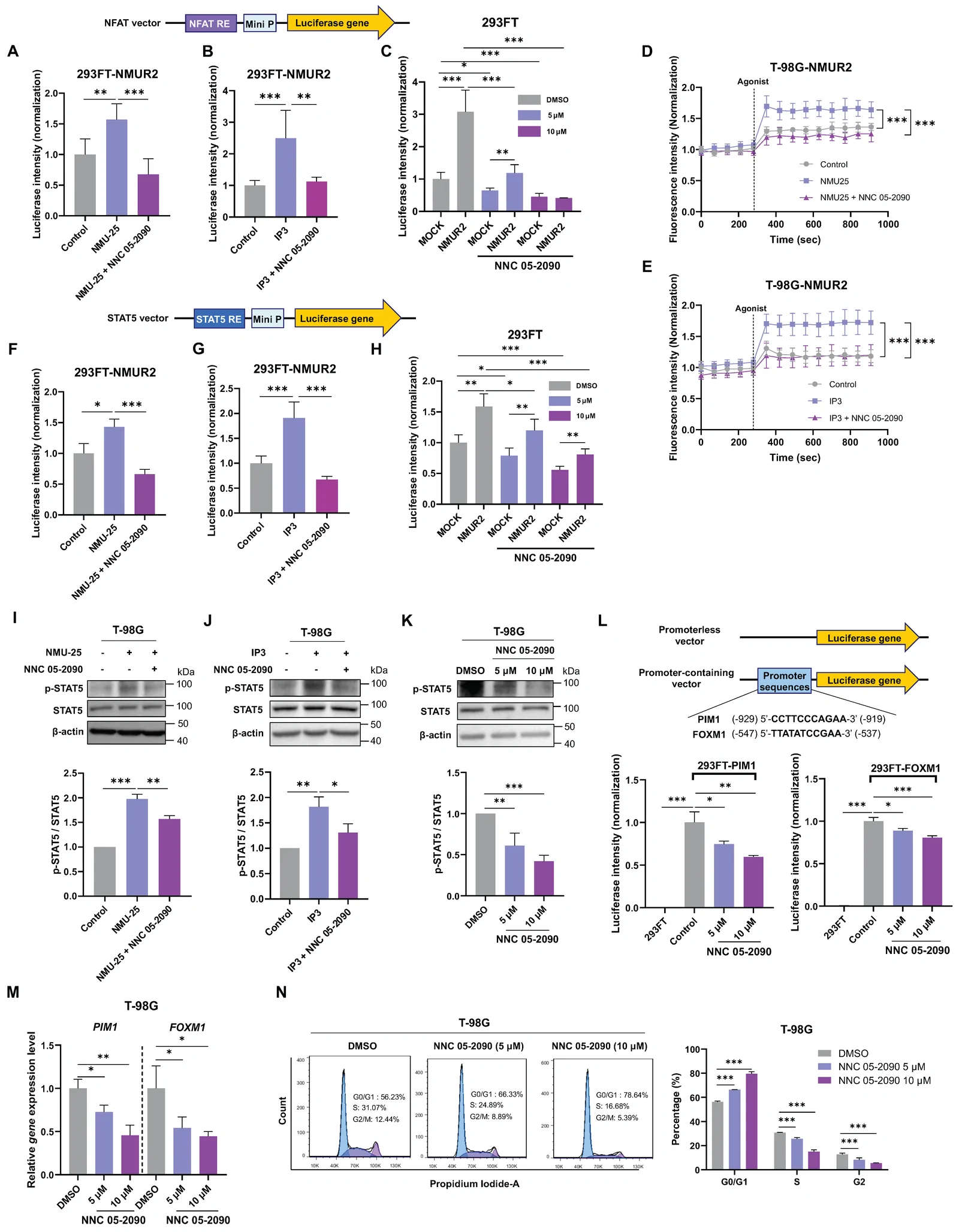
** NNC 05-2090 modulates the NMUR2/Gαq/STAT5/PIM1-FOXM1 signaling pathway.** A-C. Luciferase reporter assay using a construct containing NFAT response elements in 293FT cells. A. Luciferase activity in cells treated with NMU-25 or co-treated with NMU-25 and NNC 05-2090. B. Luciferase activity in cells treated with IP3 or co-treated with IP3 and NNC 05-2090. C. Luciferase activity in MOCK- and NMUR2-overexpressing 293FT cells treated with NNC 05-2090 at the indicated concentrations (0, 5, and 10 μM). D-E. Intracellular Ca²⁺ analysis in T-98G cells using Green FLUOFORTE Dye. D. Intracellular Ca²⁺ levels in cells treated with NMU-25 or co-treated with NMU-25 and NNC 05-2090. E. Intracellular Ca²⁺ levels in cells treated with IP3 or co-treated with IP3 and NNC 05-2090. F-H. Luciferase reporter assay using a construct containing STAT5 response elements in 293FT cells. F. Luciferase activity in cells treated with NMU-25 or co-treated with NMU-25 and NNC 05-2090. G. Luciferase activity in cells treated with IP3 or co-treated with IP3 and NNC 05-2090. H. Luciferase activity in MOCK- and NMUR2-overexpressing 293FT cells treated with NNC 05-2090 at the indicated concentrations (0, 5, and 10 μM). I-K. STAT5 phosphorylation in T-98G cells determined by western blotting. I. Phospho-STAT5 and total STAT5 levels in cells treated with NMU-25 or co-treated with NMU-25 and NNC 05-2090. J. Phospho-STAT5 and total STAT5 levels in cells treated with IP3 or co-treated with IP3 and NNC 05-2090. K. Phospho-STAT5 and total STAT5 levels in cells treated with NNC 05-2090 at the indicated concentrations (0, 5, and 10 μM). L. Luciferase reporter assay using pGL4.10-PIM1 and pGL4.10-FOXM1 promoter constructs in cells treated with NNC 05-2090. M. PIM1 and FOXM1 mRNA expression in T-98G cells 6 h after treatment with NNC 05-2090, determined by qPCR. N. Cell cycle analysis of T-98G cells treated with NNC 05-2090 at the indicated concentrations (0, 5, and 10 μM). After 12 h of treatment, cells were stained with propidium iodide and analyzed by flow cytometry to determine DNA content in G1, S, and G2/M phases. Graph shows cell cycle distribution. Data are presented as means ± SD (*P < 0.05, **P < 0.01, ***P < 0.001).

To further confirm that NNC 05-2090 modulates NMUR2 signaling, we first examined its effects on NMUR2-mediated Gαq/Ca²⁺ signaling using an NFAT luciferase reporter assay. In NMUR2-overexpressing 293FT cells, treatment with NMU-25 or IP3 increased NFAT reporter activity, whereas co-treatment with NNC 05-2090 significantly reduced this response (Fig. 6A-B). In addition, NNC 05-2090 suppressed NFAT reporter activity in a dose-dependent manner (Fig. 6C). We next assessed intracellular Ca²⁺ mobilization using a FLUOFORTE calcium assay. In NMUR2-overexpressing T-98G and 293FT cells, treatment with NMU-25 or IP3 increased intracellular Ca²⁺ levels, whereas co-treatment with NNC 05-2090 significantly attenuated this increase (Fig. 6D-E and Fig. S6A-B). These findings indicate that NNC 05-2090 inhibits NMUR2-dependent Ca²⁺ signaling.

We then investigated whether NNC 05-2090 suppresses STAT5 activation downstream of NMUR2. A STAT5-responsive luciferase reporter assay showed that NMU-25 or IP3 increased STAT5-dependent transcriptional activity, whereas co-treatment with NNC 05-2090 significantly reduced this effect in NMUR2-overexpressing 293FT cells (Fig. 6F-G). NNC 05-2090 alone also decreased STAT5 reporter activity in a dose-dependent manner (Fig. 6H). Consistent with these findings, western blot analysis showed that NMU-25 or IP3 increased phospho-STAT5 levels in T-98G cells, whereas co-treatment with NNC 05-2090 reduced STAT5 phosphorylation (Fig. 6I-J). Moreover, phospho-STAT5 levels progressively decreased with increasing concentrations of NNC 05-2090 (Fig. 6K). Together, these results indicate that NNC 05-2090 inhibits NMUR2 downstream signaling by suppressing Gαq/Ca²⁺-dependent STAT5 activation.

To determine whether inhibition of NMUR2 signaling by NNC 05-2090 affects transcription of cell cycle-associated genes, we performed promoter luciferase assays using PIM1 and FOXM1 promoter constructs in 293FT cells. NNC 05-2090 treatment significantly reduced PIM1 and FOXM1 promoter activity in a dose-dependent manner (Fig. 6L). Consistently, NNC 05-2090 decreased the expression of *PIM1* and *FOXM1* in T-98G cells (Fig. 6M). Furthermore, treatment with NNC 05-2090 increased the proportion of cells in the G0/G1 phase while reducing that in the S and G2 phases in T-98G cells (Fig. 6N). In line with these findings, NNC 05-2090 treatment reduced *CyclinB1* and* CyclinD1* expression and increased *CDKN1B* expression (Fig. S6C). Collectively, these findings indicate that NNC 05-2090 suppresses the NMUR2/Gαq/STAT5 signaling axis, resulting in downregulation of cell cycle-associated genes and induction of cell cycle arrest.

### NNC 05-2090 alone or in combination with TMZ suppresses glioma cell growth

**Figure 7:**
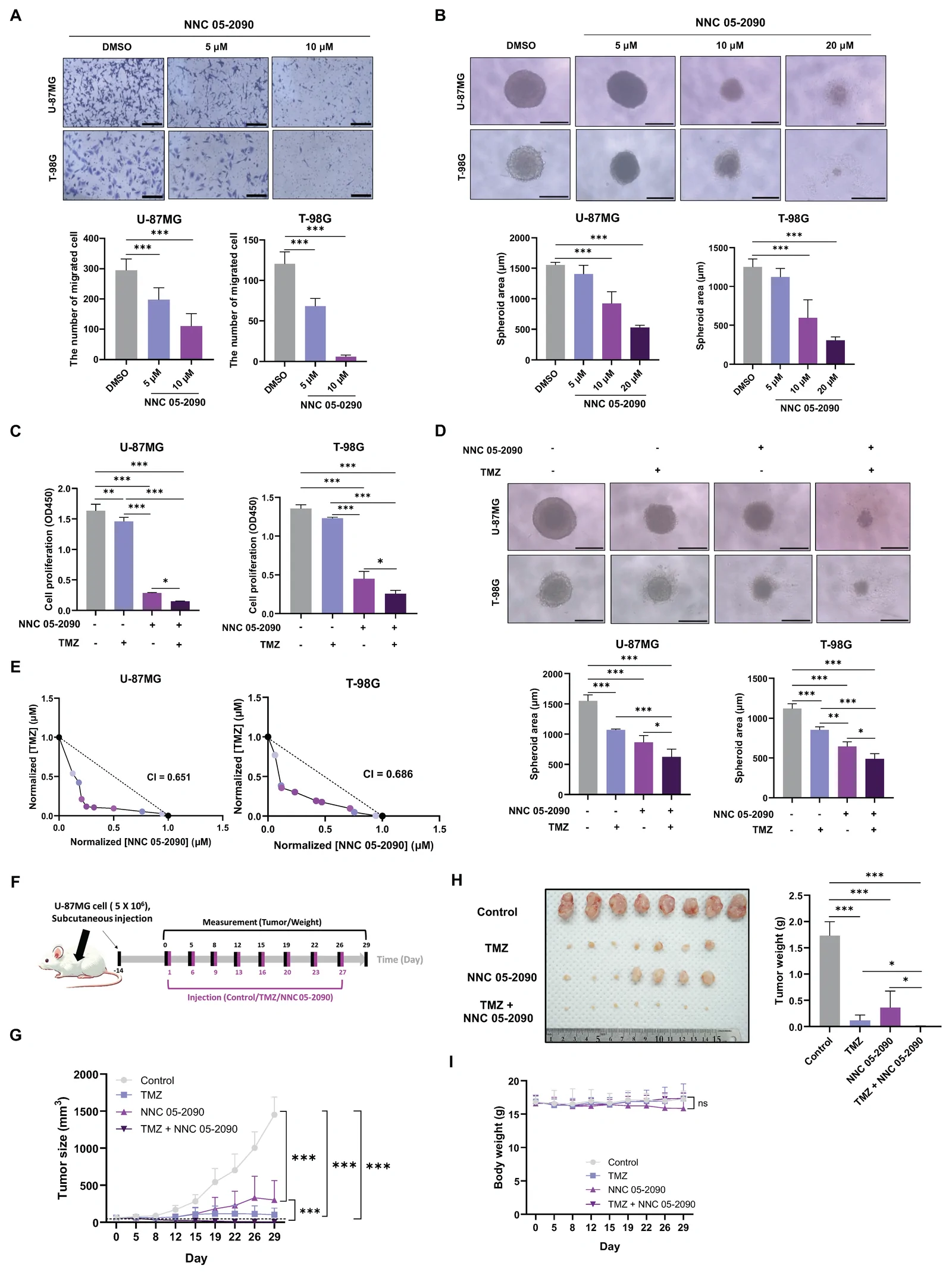
** NNC 05-2090 suppresses glioma growth *in vitro* and *in vivo*.** A. Cell migration assay in U-87MG and T-98G cells treated with DMSO or NNC 05-2090 at the indicated concentrations (5 and 10 μM) for 24 h. Cells were seeded into the upper chambers of Transwell inserts, and migrated cells were fixed and stained with crystal violet. Representative images and quantification of migrated cells are shown. Scale bar, 500 μm. B. Representative images of U-87MG and T-98G spheroids following treatment with the indicated concentrations of NNC 05-2090. Scale bar, 1000 μm. C. Cell proliferation in U-87MG (left) and T-98G (right) cells treated with NNC 05-2090 (10 μM), temozolomide (TMZ, 1 mM), or the combination of NNC 05-2090 and TMZ. D. Representative images of U-87MG and T-98G spheroids treated with NNC 05-2090 (10 μM), TMZ (1 mM), or the combination of NNC 05-2090 and TMZ. Scale bar, 1000 μm. E. Combination index (CI) analysis of NNC 05-2090 and TMZ treatment in U-87MG and T-98G cells. F. Schematic diagram of the animal experimental timeline. U-87MG cells were subcutaneously injected into the flanks of NOG mice. Mice were assigned to four groups: control, TMZ (30 mg/kg), NNC 05-2090 (50 mg/kg), and TMZ + NNC 05-2090. G. Tumor growth curves of xenograft-bearing mice in each treatment group. Tumor volumes were measured twice per week. Data are presented as mean ± SEM (n = 7 per group). H. Representative images of excised tumors from each treatment group and tumor weight quantification at the end of the experiment. Tumors were weighed after excision. I. Body weight changes in mice during the treatment period. Data are presented as mean ± SEM. *P < 0.05, **P < 0.01, ***P < 0.001.

To evaluate the therapeutic potential of NNC 05-2090 in glioma, U-87MG and T-98G cells were treated with increasing concentrations of NNC 05-2090, and malignant phenotypes were assessed. NNC 05-2090 treatment significantly reduced cell migration and sphere-forming ability while increasing apoptotic cell death in a dose-dependent manner (Fig. 7A-B and Fig. S7A). These findings indicate that NNC 05-2090 exerts anti-tumor effects in glioma cells.

We next examined whether NNC 05-2090 enhances the anti-glioma activity of temozolomide (TMZ). Combined treatment with NNC 05-2090 and TMZ significantly reduced cell proliferation and sphere formation and increased apoptosis compared with either agent alone in GBM cell lines (Fig. 7C-D and Fig. S7B). To quantitatively assess the interaction between NNC 05-2090 and TMZ, combination index (CI) analysis was performed as previously described 32. CI values were < 1 in both U-87MG and T-98G cells, indicating a synergistic interaction between NNC 05-2090 and TMZ (Fig. 7E).

To validate these findings *in vivo*, we transplanted glioma tumors subcutaneously in mice and treated them with TMZ alone, NNC 05-2090 alone, or a combination of TMZ and NNC 05-2090 (Fig. 7F). Significant reductions in tumor size were observed in the TMZ or NNC 05-2090 monotherapy groups compared with the control group. Furthermore, the group treated with combined TMZ and NNC 05-2090 therapy demonstrated enhanced tumor growth inhibition relative to the single-treatment groups (Fig. 7G). After 29 days of treatment, tumors were excised and weighed. Tumor weights were significantly decreased in the TMZ or NNC 05-2090 monotherapy groups compared with the control group, with the combination therapy group exhibiting the most pronounced reduction (Fig. 7H).

To assess treatment-related toxicity, body weight, serum chemistry, and histopathology were examined. No significant differences in body weight or serum levels of AST, ALT, CK, and BUN were observed between control and NNC 05-2090-treated mice (Fig. 7I and Fig. S7C). In addition, H&E staining of major organs revealed no detectable treatment-related histopathological abnormalities (Fig. S7D). Together, these findings demonstrate that NNC 05-2090 suppresses glioma growth both alone and in combination with TMZ, with no evident *in vivo* toxicity under the conditions tested.

## Discussion

Despite advances in multimodal therapy, gliomas remain among the most lethal human cancers. Temozolomide (TMZ) remains the principal chemotherapeutic agent for glioma, and bevacizumab has also been used in selected clinical settings 33. In addition, molecularly targeted agents such as savolitinib, a c-MET inhibitor, and ivosidenib, an IDH1 inhibitor, have been developed for specific patient subsets 34, 35. Nevertheless, therapeutic efficacy remains limited, and the median survival of patients with malignant glioma is still less than 15 months. Major clinical challenges include limited blood-brain barrier (BBB) penetration, marked intratumoral heterogeneity, resistance to standard therapies such as TMZ and radiotherapy, and the diffuse infiltrative growth pattern that prevents complete surgical resection 36. Furthermore, the immunosuppressive tumor microenvironment constrains the efficacy of immunotherapy, and robust biomarkers for treatment stratification remain limited 37. Together, these challenges underscore the need for new therapeutic strategies, improved drug delivery approaches, biomarker-guided patient selection, and a deeper mechanistic understanding of glioma biology.

In the present study, we identified NMUR2 as a previously underappreciated regulator of glioma progression. NMUR2 was highly expressed in human glioma tissues and promoted malignant phenotypes in glioma cells, including proliferation and migration. Mechanistically, NMUR2 activated Gαq-dependent signaling, resulting in intracellular Ca²⁺ mobilization and subsequent STAT5 phosphorylation. Because STAT5 functions as a transcriptional activator, we further examined its downstream transcriptional program and found that NMUR2 signaling enhanced the expression of the cell cycle-associated genes PIM1 and FOXM1 through STAT5-dependent promoter activation. These findings position NMUR2 as an upstream regulator of a pro-tumorigenic signaling cascade involving the Gαq/Ca²⁺/STAT5/PIM1 and FOXM1 axis in glioma.

The involvement of Gαq signaling in STAT5 activation is supported by previous studies of other GPCRs. For example, oxytocin receptor (OXTR) signaling in the mammary gland has been associated with altered STAT5 phosphorylation and regulation of lactation-related functions 38. Likewise, GPR54 activation has been reported to enhance mTOR, ERK1/2, and STAT5 signaling, thereby promoting β-casein synthesis 39. Although these studies were not conducted in cancer models, they provide mechanistic support for the concept that GPCR-mediated Gαq signaling can influence STAT5 activation. Our findings extend this concept to glioma and suggest that NMUR2 engages this pathway to promote tumor cell proliferation. However, the precise molecular events linking Ca²⁺ mobilization to STAT5 phosphorylation in glioma cells remain to be defined and warrant further investigation.

Our findings are also consistent with previous reports implicating the NMU-NMUR2 axis in tumor progression. In endometrial cancer, NMUR2 activation has been shown to alter the expression of adhesion-related molecules, including CD44 and integrin α1, thereby promoting tumor progression 22. In colorectal cancer, NMUR2 signaling induced by NMU enhances invasive phenotypes and increases integrin subunit expression 23. Beyond receptor-level regulation, NMU itself is frequently overexpressed in multiple malignancies, including colorectal, lung, and liver cancers, where it contributes to proliferation, motility, invasion, and therapeutic resistance 27, 40-43. Disruption of this signaling axis, either by inhibiting NMU or blocking NMUR2, has been shown to attenuate aggressive tumor phenotypes and restore treatment sensitivity 44, 45. Our study expands these observations by showing that, in glioma, NMUR2 regulates a STAT5-dependent transcriptional program linked to cell cycle progression.

STAT5 is well known to undergo dimerization and nuclear translocation following phosphorylation, where it functions as a transcription factor to regulate genes involved in proliferation, survival, and differentiation. Aberrant STAT5 activation has been implicated in the progression of multiple cancer types 46-48. Although STAT5 can regulate a broad range of target genes, our study focused on PIM1 and FOXM1, two genes with well-established roles in cell cycle progression and oncogenesis. The relationship between STAT5 and PIM1 has been documented in hematologic malignancies, including precursor T-cell acute lymphoblastic leukemia (T-ALL) and T-cell lymphoblastic lymphoma (T-LBL), in which JAK/STAT pathway activation increases PIM1 expression and contributes to disease progression 49, 50. In contrast, evidence linking STAT5 to FOXM1 regulation has been limited. Previous work showed that FOXM1 can be regulated in a STAT3-dependent manner in glioblastoma, contributing to radioresistance 51. Our findings identify FOXM1 as a downstream target of NMUR2-STAT5 signaling and suggest a previously unrecognized connection between GPCR signaling and FOXM1-mediated cell cycle control in glioma. Thus, NMUR2 signaling appears to promote glioma progression, at least in part, by activating a STAT5-dependent transcriptional program centered on PIM1 and FOXM1.

Although our study focused on PIM1 and FOXM1 as downstream targets of NMUR2-STAT5 signaling, these genes likely represent selected components of a broader cell cycle-associated transcriptional program rather than the only genes regulated by this pathway. RNA-sequencing analysis showed that cell cycle-related pathways were prominently altered following NMUR2 depletion, and subsequent validation experiments further supported the involvement of NMUR2 in cell cycle regulation. PIM1 and FOXM1 were selected for further investigation not solely on the basis of fold-change magnitude, but also because of their established roles in cell cycle progression, tumor growth, and therapeutic resistance, as well as the presence of predicted STAT5-binding elements within their promoter regions. Although FOXM1 was not the most prominently altered gene in the RNA-seq dataset, its well-documented role in glioma biology, including cell cycle progression, stemness, and treatment resistance, supports its biological relevance in this context. In addition, changes in other cell cycle-associated genes, including Cyclin B1, Cyclin D1, and CDKN1B, further support the broader effect of NMUR2 signaling on cell cycle control. Thus, PIM1 and FOXM1 should be regarded as functionally validated mediators within the NMUR2-STAT5-dependent transcriptional network, and future studies will be needed to define the full spectrum of STAT5-regulated genes downstream of NMUR2 in glioma.

Both PIM1 and FOXM1 are established oncogenic regulators that contribute to cancer progression by promoting cell survival, proliferation, and therapeutic resistance. PIM1, a constitutively active serine/threonine kinase, supports tumor cell viability by suppressing apoptosis and promoting cell cycle progression through targets such as CDC25A and p21 52, 53. FOXM1, a transcription factor frequently overexpressed in aggressive cancers, promotes both G1/S and G2/M transitions by inducing mitotic regulators including cyclin B1, PLK1, and Aurora B kinase 54, 55. FOXM1 has also been implicated in stemness, metastasis, and chemoresistance across multiple tumor types 56. In this context, our findings suggest that NMUR2 functions as an upstream regulator that converges on both PIM1- and FOXM1-dependent oncogenic programs. Targeting NMUR2 may therefore provide an upstream strategy to suppress multiple cell cycle-promoting pathways simultaneously.

An important translational aspect of this study is the identification of NNC 05-2090 as a candidate NMUR2 antagonist. NNC 05-2090 has previously been described as a GABA uptake inhibitor with activity in neurological contexts 29, 30. Using the PRESTO-Tango assay, we screened a 6,331-compound drug-repurposing library and identified NNC 05-2090 as a compound that inhibits NMUR2 signaling. Computational modeling predicted that NNC 05-2090 binds within a ligand-binding pocket similar to that occupied by NMU, supporting a competitive mode of interaction. This prediction was further supported by CETSA, which demonstrated increased thermal stability of NMUR2 in the presence of NNC 05-2090, consistent with direct target engagement. Notably, the inhibitory activity of NNC 05-2090 was lower against other tested GPCRs, including EDNRA, GPR87, GPR161, GPR55, and GPR91, suggesting relative selectivity for NMUR2. Functionally, NNC 05-2090 suppressed NMUR2-mediated Gαq/Ca²⁺/STAT5 signaling, reduced PIM1 and FOXM1 expression, induced cell cycle arrest, and inhibited glioma growth *in vitro* and *in vivo*. Furthermore, the combination of NNC 05-2090 with TMZ produced synergistic anti-tumor effects, supporting its potential therapeutic value.

Several limitations of this study should be acknowledged. First, although our data support a role for Ca²⁺ signaling in STAT5 activation downstream of NMUR2, the intermediate signaling mechanisms were not fully defined. Second, while computational docking and CETSA suggest a direct interaction between NNC 05-2090 and NMUR2, the precise binding mode remains to be characterized by additional structural studies. Third, although in vivo experiments showed anti-tumor efficacy without overt toxicity, the BBB penetration and pharmacokinetic properties of NNC 05-2090 were not comprehensively evaluated. Finally, given the molecular and cellular heterogeneity of glioma, further validation using additional patient-derived and orthotopic models will be necessary to establish the broader applicability of our findings.

## Conclusion

**Figure 8:**
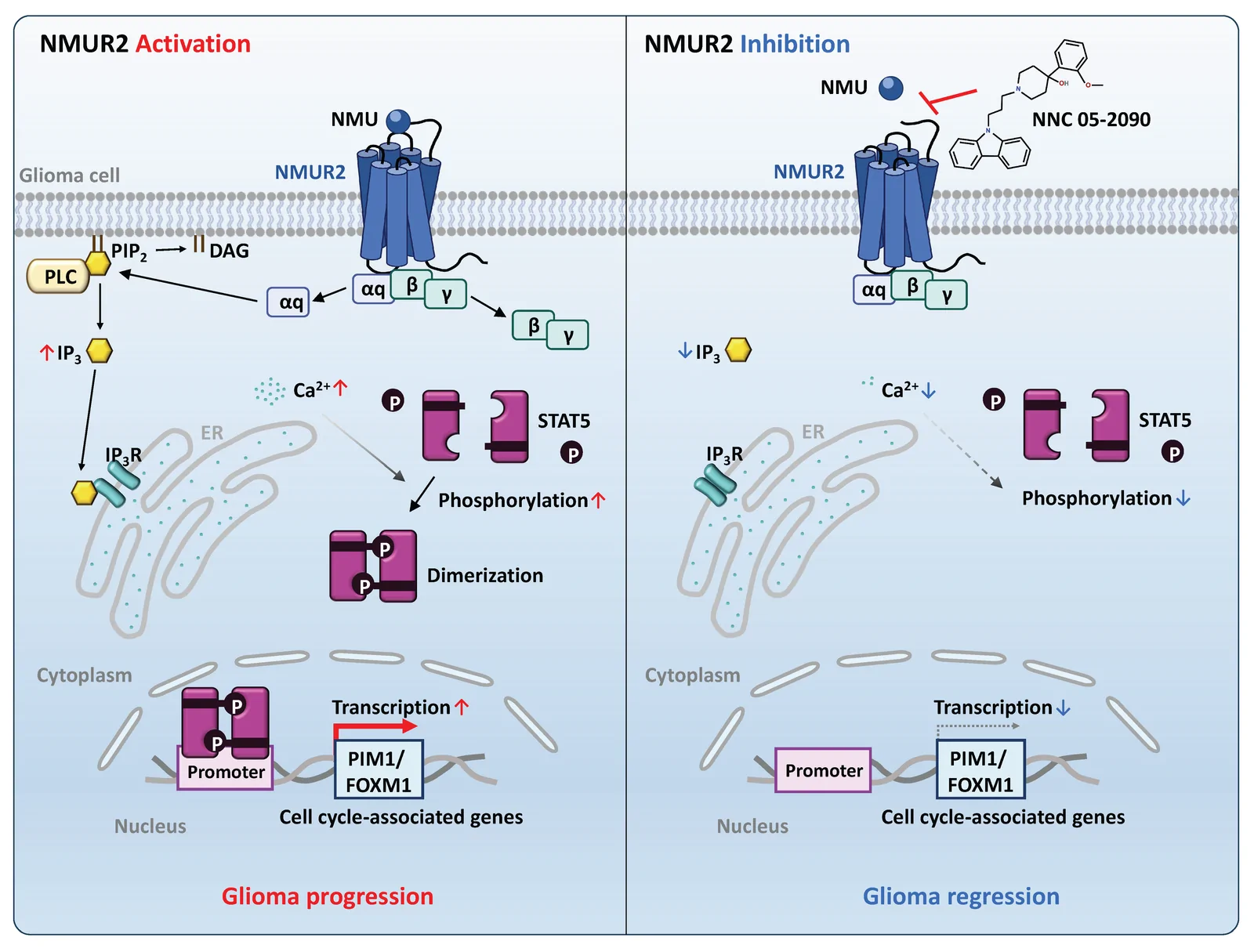
** Discovery of a novel NMUR2 signaling pathway and its inhibition by NNC 05-2090 in glioma.** The proposed oncogenic mechanism of NMUR2 signaling involves activation of the Gαq-STAT5-PIM1/FOXM1 pathway in glioma. When NMUR2 is overexpressed or activated, it triggers Gαq-mediated IP_3_ production, which subsequently induces Ca²⁺ release from the endoplasmic reticulum (ER). Elevated intracellular Ca²⁺ promotes STAT5 phosphorylation, and phosphorylated STAT5 then translocates into the nucleus and induces the expression of cell cycle-related genes *PIM1* and *FOXM1*, thereby promoting glioma progression (left). In contrast, NNC 05-2090, a novel NMUR2-specific antagonist identified in this study, binds to NMUR2 and inhibits its activation. This leads to inhibition of downstream signaling, ultimately suppressing glioma progression (right).

In conclusion, our findings identify NMUR2 as a potential therapeutic target in glioma. NMUR2 promoted the proliferation of human glioma cells by activating Gαq-dependent signaling, leading to intracellular Ca²⁺ mobilization, STAT5 phosphorylation, and transcriptional upregulation of the cell cycle-associated genes PIM1 and FOXM1. Through this signaling cascade, NMUR2 contributed to glioma progression. We further identified NNC 05-2090 as a candidate antagonist of NMUR2. Treatment with NNC 05-2090 suppressed NMUR2-mediated signaling, downregulated PIM1 and FOXM1, induced cell cycle arrest, and inhibited glioma growth *in vitro* and *in vivo* (Fig. 8). Notably, combination treatment with NNC 05-2090 and TMZ produced synergistic anti-tumor effects. Although further studies will be required to establish its clinical feasibility, these findings support pharmacological inhibition of NMUR2 as a potential therapeutic strategy for glioma.

## Materials and Methods

### Cell culture

The experimental cell lines were purchased from the Korean Cell Line Bank (Seoul, South Korea) and maintained at 37°C in a 5% CO_2_ incubator using appropriate growth media for each cell line. U-87MG cells (RRID: CVCL_0022) were cultured in Minimum Essential Medium (MEM; Welgene, Gyeongsan-si, Republic of Korea) supplemented with 10% Fetal Bovine Serum (FBS; Welgene) and 1% Antibiotic-Antimycotic (100X) (Gibco, Waltham, MA, USA). T-98G cells (RRID: CVCL_0556) were cultured in Dulbecco's Modified Eagle's Medium (DMEM; Welgene) with 10% FBS and 1% Antibiotic-Antimycotic (100X). The U-87MG and T-98G cell lines, which continue to be widely used in recent glioblastoma research, were employed in this study for their relevance to glioma biology. 293FT cells (RRID: CVCL_6911) were cultured in DMEM with 10% FBS and 1% Antibiotic-Antimycotic (100X). The HTLA (a HEK293 cell line (RRID: CVCL_0045) stably expressing a tTA-dependent luciferase reporter and a β-arrestin2-TEV fusion gene) cell line, which was specifically used for drug screening in this study, was cultured in DMEM with 10% FBS, 1% Antibiotic-Antimycotic (100X) and 1% GlutaMAX (Gibco). All cell lines were authenticated by the Korean Cell Line Bank and were confirmed to be free of mycoplasma contamination before use in experiments.

### Chemicals

A drug-repositioning library was purchased from MedChem Express, Tocris, and Selleckchem for *in vitro* drug screening. NNC 05-2090 (Abcam plc, Cambridge, UK), Neuromedin U-25 (NMU-25; Bachem AG, Bubendorf, Switzerland), and Temozolomide (LKT Laboratories, Inc., St. Paul, MN, USA) were used for *in vitro* experiments, while Temozolomide (Sigma-Aldrich, Burlington, MA, USA) was used for *in vivo* experiments. Additionally, IP_3_ (Avanti Polar Lipids, Alabaster, AL, USA), Xestospongin C (Santa Cruz Biotechnology, Dallas, TX, USA), Pimozide (Sigma-Aldrich, Burlington, MA, USA) were also used.

### Tissue microarray

Paraffin-embedded tissue microarray slide glasses (GL807a and GL208a) were purchased from TissueArray.Com (Derwood, MD, USA). For immunohistochemical staining of NMUR2, the tissue sections were deparaffinized, rehydrated, and subjected to antigen retrieval using citric acid and trisodium citrate. Endogenous peroxidase activity was quenched by incubating the sections with 3% hydrogen peroxide at room temperature for 5 minutes, followed by three washes with 0.05% TBST. The sections were then blocked with 5% normal goat serum at room temperature for 1 hour and incubated overnight at 4°C with anti-NMUR2 antibody (1:100; Novus Biologicals, LLC, Centennial, Colorado, USA). After washing, the sections were incubated with the ImmPRESS® HRP anti-rabbit IgG polymer kit (Vector Laboratories, Inc., Burlingame, CA, USA) for 1 hour at room temperature. Color development was achieved by applying the ImmPACT DAB substrate kit (Vector Laboratories, Inc.) for 1 minute. Nuclear counterstaining was performed using hematoxylin (Agilent Technologies, Inc., Santa Clara, California, USA). Finally, the slides were dehydrated and sealed using a mounting solution.

### Vectors

The pcDNA3.1 zeo(+) vector (Thermo Fisher Scientific, Waltham, MA, USA) and the pIRES-EGFP vector (TAKARA Bio Inc., Kusatsu, Shiga, Japan) were used to insert the coding region of the gene of interest. The pGL4.30[luc2P/NFAT-RE/Hygro] (Promega Corporation, Madison, WI, USA) and the pGL4.52[luc2P/STAT5 RE/Hygro] (Promega Corporation) vectors were used for the luciferase reporter assay. Additionally, the pGL4.10[luc2] vector (Promega Corporation) was employed for the promoter assay.

### Transfection

For overexpression experiments, the coding sequence (CDS) of NMUR2 was subcloned into the pIRES-EGFP vector. The NMUR2 CDS was amplified by PCR. The NMUR2 CDS primers are listed in Supplementary Table 1. These primers contained restriction sites for EcoRI and SacII to facilitate cloning into the pIRES-EGFP vector. For knockdown experiments, siRNA sequences were used listed in Supplementary Table 1. Glioma cell lines were plated at a density of 50-60% confluency one day prior to transfection. Transfection was performed using Lipofectamine 2000 (Thermo Fisher Scientific) for the overexpression vector or Lipofectamine RNAiMAX (Thermo Fisher Scientific) for siRNA, following the manufacturer's protocols. After 24-48 h of incubation in a humidified incubator at 37°C with 5% CO_2_, subsequent experiments were conducted.

### RNA isolation and quantitative real-time PCR

Total RNA was isolated from cells using NucleoZol reagent (Macherey-Nagel GmbH & Co. KG, Düren, Germany) according to the manufacturer's instructions. CellScript cDNA Master Mix (Cellsafe Co., Ltd., Yongin, Republic of Korea) was used for the synthesis of cDNA. Quantitative real-time PCR was performed using SYBR Green PCR Master Mix (Applied Biosystems, Waltham, MA, USA) on the StepOne Plus Real-time System. The PCR amplification was carried out on every sample using an equivalent quantity of cDNA template. Following an initial step in the thermal cycler for 10min at 95℃, the PCR amplification was proceeded for 40 cycles of 15 s at 95°C, 30 s at 60°C ,1 min at 72°C, and completed by melting curve analysis to confirm the specificity of the PCR products. All experiments were conducted with a minimum of three repetitions. Transcript levels were normalized to GAPDH levels. The primer used in this experiment are listed in Supplementary Table S1.

### Western blot

Cells were lysed in RIPA lysis buffer (Thermo Fisher Scientific) with Halt Protease and Phosphatase inhibitor Cocktail (Thermo Fisher Scientific). The protein lysates were centrifuged at 13,000 rpm at 4°C for 20 min, and bicinchoninicacid assay (BCA) was used for quantitation of protein lysates. Then, those lysates were boiled for 95°C, 5 min. Lysates were separated using SDS-polyacrylamide gel electrophoresis (PAGE) and transferred to a PVDF membranes (Millipore). The membranes were blocked with The 4% BSA in TBST with 0.1% Tween-20 for 1 h at 25°C and incubated with primary antibodies at 4°C overnight using anti-NMUR2 (Invitrogen, Waltham, MA, USA) 1:1000, anti-phospho STAT5a/b (R&D Systems, Minneapolis, MN, USA) 1:1000, anti-STAT5a/b (R&D Systems) 1:1000, anti-PIM1 (Cell Signaling Technology, Danvers, MA, USA) 1:1000, anti-FOXM1 (Cell Signaling Technology) 1:1000, anti-β-actin (AbClon Inc., Seoul, Republic of Korea) 1:3000. Secondary antibody was added and incubated for 1 h at 25°C using anti-Rabbit IgG HRP-linked antibody (Cell Signaling Technology). Proteins were detected by chemiluminescence method using Amersham™ ECL (Cytiva, Marlborough, MA, USA) and quantified using iBright™ CL1500 Imaging System (Invitrogen).

### Phosphokinase array

The Proteome Profiler Human Phospho-Kinase Array Kit (R&D Systems) was used for the phosphokinase assay according to the manufacturer's instructions. T-98G cells were transfected with either negative control siRNA (siNC) or siRNAs targeting NMUR2 (siNMUR2-1 and siNMUR2-2) using Lipofectamine™ RNAiMAX (Thermo Fisher Scientific). After 48 h, the cells were lysed using Lysis Buffer 6 and incubated for 30 min at 4°C on a rocking shaker. The lysates were centrifuged at 14,000 × g for 5 min at 4°C, and the supernatants were transferred to new Eppendorf tubes. Protein concentrations were measured using the Pierce™ BCA Protein Assay Kit (Thermo Fisher Scientific). Membranes were incubated overnight at 4°C on a rocking shaker with 2 ml of diluted cell lysates. Following incubation, Detection Antibody Cocktail A or B (DAC-A or DAC-B) was added to the membranes, followed by 1 mL of diluted Streptavidin-HRP. Protein detection was performed using chemiluminescence with Amersham™ ECL. Spot densities of the phosphokinase array were analyzed using ImageJ software, with intensity values normalized to the average of the negative control.

### Cell proliferation assay

An equal number of cells were seeded into each well of 96-well plates. Cell viability was measured using the Cell Counting Kit-8 (CCK-8; Dojindo Molecular Technologies, Inc., Dojimachi, Yamaguchi, Japan) reagent. For the NMUR2 overexpression and knockdown experiments, U-87MG and T-98G cells were seeded at 3,000 cells per well, and cell proliferation was assessed at 24-, 48-, 72-, and 96-hour post-transfection. For NMUR2 activation, U-87MG and T-98G cells were treated with NMU-25 to activate NMUR2, and cell proliferation was assessed at 24-, 48-, and 72-hour post-treatment. Following CCK-8 reagent treatment, the plates were incubated for 1 h at 37°C, and optical density (OD) was measured at 450 nm using a TecanSunrise microplate reader. For the IC_50_ determination of NNC 05-2090, U-87MG cells were seeded at 8,000 cells per well, and T-98G cells were seeded at 5,000 cells per well. The cells were treated with varying concentrations of NNC 05-2090 (0, 1.25, 2.5, 5, 10, 20 μM) for 48 h. After CCK-8 reagent treatment, the plates were incubated for 1 h at 37°C, and OD values were measured at 450 nm using a TecanSunrise microplate reader.

### Cell apoptosis assay

An equal number of cells from each cell line were evenly distributed into each well of a white 96-well cell culture plate. Apoptosis was assessed 24 h later using the Caspase-Glo 3/7 Assay System (Promega Corporation). After treatment with Caspase-Glo 3/7, the plate was incubated in the dark at 25°C for 30 min, and luminescence was measured using a Berthold Centro XS3.

### Transwell migration assay

Transwell migration assay was performed using transwell with 8 μM 24-well 'insert' (Corning Inc., Corning, NY, USA). The cells were suspended in 500 μL of serum-free medium and seeded in the upper chamber, and 500 μL medium was added to the lower chamber. After 24 h, media in both the upper insert and lower chambers were removed. Cells that had migrated into the lower chamber through the 8-μM pore membrane were fixed with 4% formaldehyde and 100% methanol and were stained by 0.1% crystal violet solution (Merck KGaA, Darmstadt, Germany), and then, the migrated cells were visualized using a microscope.

### Cell cycle analysis

The cell cycle analysis was performed using BD Cycletest™ Plus DNA Reagent Kit (BD Biosciences, Franklin Lakes, NJ, USA) in accordance with the manufacturer's instructions. T-98G cells were cultured in 6-well plates and subsequently subjected to either transfection or drug treatment. Following this, the cells were harvested and centrifuged at 300 × g for 5 min at 25°C. After removing the supernatant, 1 mL of buffer solution was added to resuspend the cells, which were then centrifuged again at 300 × g for 5 min. This process was repeated twice, and the cell concentration was adjusted to 1 × 10^6^ cells/ml using buffer solution before centrifugation at 400 × g for 5 min. After carefully removing all supernatants, Solution A (trypsin buffer) and Solution B (trypsin inhibitor and RNase buffer) were sequentially added at 25°C for 10 min. Subsequently, 200 μL of cold (2-8°C) Solution C (PI stain solution) was added, and the cells were incubated at 4°C in the dark for 10 min. Finally, the stained cells were subjected to cell cycle analysis using the BD FACSVerse™ Cell Analyzer (BD Biosciences).

### Spheroid assay

Cell spheroids were cultured in a 96-well Ultra Low Attachment plate (Corning Inc). U-87MG cells were grown in Alpha MEM (Welgene) supplemented with 10% FBS and 1% Antibiotic-Antimycotic (100X), while T-98G cells were cultured in DMEM/F-12 (Welgene) with the same supplements. The seeding density was 600 cells/well, and the cells were maintained at 37°C with 5% CO_2_ for 4 days. After this period, NNC 05-2090 and TMZ were treated. Both drugs were added every three days to maintain the desired concentration, with two total treatments for each drug.

### Construction of PRESTO-TANGO vector

The NMUR2 TANGO sequence and the pIRES-EGFP vector were inserted into the pcDNA3.1-zeocin (+)-HA tag vector to construct the pIRES-EGFP-NMUR2 TANGO vector. The NMUR2 TANGO sequence, containing the tTA transcription factor, was derived from the NMUR2 TANGO vector (plasmid #66447). The NMUR2 TANGO sequence is listed in Supplementary Table S1. To insert the NMUR2 TANGO sequence into the pcDNA3.1(+) vector, restriction enzymes XheI and XbaI were used. For the subsequent insertion of the pIRES-EGFP vector into the pcDNA3.1(+) vector, restriction enzymes XbaI, EcoRI, and HapI were utilized in conjunction with T4 DNA polymerase.

### PRESTO-TANGO (β-arrestin) assay

HTLA cells (PRESTO-Tango reporter cells), a modified HEK293 cell line engineered to express a luciferase reporter under tTA control and a fusion gene consisting of β-arrestin and TEV, were utilized for the β-arrestin assay. The cells were transfected with a plasmid containing an optimized sequence of NMUR2 (pIRES-EGFP-NMUR2) and subsequently sorted based on EGFP intensity using Fluorescence-activated Cell Sorting (FACS). On day 1, cells were seeded at a density of 2 × 10⁴ cells per well in white 96-well plates. On day 2, the cells were treated for 6 h with a 6,331-compound drug-repurposing library. NMU-25 (20 nM) as the agonist for NMUR2, while cycloheximide (10 μM) was used as a control inhibitor. β-arrestin activity was quantified using the One-Glo solution (Promega Corporation). Following One-Glo solution treatment, the white 96-well plates were incubated at 25°C for 1 min and 30 sec. Luminescence was then measured using a Berthold Centro Xs³ luminometer.

### *In silico* prediction of NMUR2-NNC 05-2090 interaction

The interaction between NMUR2 and NNC 05-2090 was evaluated using two *in silico* approaches. First, blind docking analysis was performed using CB-Dock2 57 with the NMUR2 structure (PDB ID: 7XK8) 31. Second, AI-based binding prediction was additionally conducted using the Pharmaco-Net platform powered by CALICI (https://calici.co.kr/). For Pharmaco-Net analysis, the NMUR2 structure was uploaded in PDB format and NNC 05-2090 was used as the query ligand. The predicted interaction was assessed based on the output parameters provided by each platform, including vina score and cavity information from CB-Dock2, and binding energy and binding affinity from Pharmaco-Net.

### Luciferase reporter assay

Luciferase intensity was measured using the Dual-Luciferase Reporter Assay System (Promega Corporation,) according to the manufacturer's protocol. First, 293FT cells were transfected with luciferase reporter gene vectors (pGL4.30, pGL4.52, and pGL4.10[luc2]) using Lipofectamine 2000 (Thermo Fisher Scientific) following the manufacturer's instructions. Co-transfection with a pRL Renilla Luciferase Control Reporter Vector (Promega Corporation) was performed as an internal control in 293FT cells. After 48 h of transfection, the cells were seeded in a white 96-well plate at a density of 5 × 10^4^ cells per well and incubated overnight at 37°C in a 5% CO₂ incubator. Following overnight incubation, the culture medium was replaced with fresh medium, and the cells were incubated for an additional 6 h. The culture medium was then removed, and the cells were lysed using 50 µL of Passive Lysis Buffer provided in the kit. The plates were placed on a shaker for 30 min to ensure complete lysis. Firefly luciferase activity was measured by adding 20 µL of Luciferase Assay Reagent II to each well, followed by immediate luminescence measurement using a Berthold Centro Xs3 luminometer. Renilla luciferase activity was subsequently measured in the same wells after the addition of 20 µL of Stop & Glo Reagent. Firefly luciferase activity was normalized to Renilla luciferase activity to account for variations in transfection efficiency. The normalized luciferase activity (Firefly/Renilla ratio) was expressed as a fold change relative to the control group.

### Promoter assay

The promoter regions of PIM1 and FOXM1 (-1,000 to +100 bp) were amplified from genomic DNA by PCR. The primers used in this experiment are listed in Supplementary Table 1. The primers included restriction sites for NheI and XhoI for cloning into the pGL4.10 [luc2] vector. The sequence and orientation of the insert were confirmed by Sanger sequencing. 293FT cells were co-transfected with 2000 ng of the pGL4.10-promoter construct and 40 ng of the pRL Renilla Luciferase Control Reporter Vector using Lipofectamine™ 2000, following the manufacturer's protocol. After 48 h of transfection, the cells were seeded into a white 96-well plate at a density of 5 × 10^4^ cells per well in DMEM supplemented with 10% FBS and incubated overnight at 37°C in a 5% CO₂ incubator. The promoter activities of PIM1 and FOXM1 were assessed using the Dual-Luciferase Reporter Assay System according to the manufacturer's instructions. Firefly and Renilla luciferase activities were measured using a Berthold Centro Xs3 luminometer. Relative promoter activity was calculated as the ratio of Firefly to Renilla luciferase activity.

### Calcium mobility assay

Intracellular calcium levels were measured using the FLUOFORTE™ Calcium Assay Kit (Enzo Biochem, Inc., Farmingdale, NY, USA) following the manufacturer's instructions. Cells were seeded into black, clear-bottom 96-well plates at a density of 4 × 10^4^ cells/well and incubated overnight at 37°C in a 5% CO₂ incubator. The culture medium was then removed, replaced with fresh medium, and incubated for an additional 6 h. After discarding the medium, 50 µL of FLUOFORTE™ dye was added to each well. Plates were incubated at 37°C for 45 min, protected from light. Fluorescence intensity was measured using a BioTek Multi-Mode Microplate Reader (excitation: 490 nm, emission: 525 nm). Baseline fluorescence was recorded for 5 min prior to stimulation. A final concentration of 1 µM NMU-25 was added to each well, and calcium flux was monitored for 10 min. The change in fluorescence intensity (F/F₀) was calculated, where F₀ represents the baseline fluorescence, and F is the maximum fluorescence intensity observed.

### Cellular thermal shift assay (CETSA)

Samples were prepared from HTLA-NMUR2 cells exposed to either the control or the drug. For each group, 2 × 10^7^ cells were seeded in a 150-mm cultured dish. After 24 h of culturing, the cells were pretreated with either DMSO or 30 μM NNC 05-2090 for 1 h in the CO_2_ incubator at 37°C, followed by washing with Dulbecco's Phosphate-Buffered Saline (DPBS, Welgene), treatment with TrypLE™ Express Enzyme (Thermo Fisher Scientific), and then harvested into 15-ml conical tubes. The samples were centrifuged at 300 × g for 5 min at 25°C to pellet the cells, after which all culture medium was carefully removed and then discarded. The cell pellets were then gently resuspended in 15 mL of PBS and centrifuged at 300 × g for 5 min at 25°C to pellet the cells again. The supernatant was carefully removed and then discarded. Subsequently, 600 μL of PBS supplemented with protease inhibitors was added to each respective tube, and the cell pellets were carefully resuspended. Each cell suspension was aliquoted into five different 0.2-ml PCR tubes with 100 µL in each and was heated at 50°C, 55°C, and 60°C for 3 min. The samples were then transferred to Eppendorf tubes and subjected to five freeze-thaw cycles using liquid nitrogen and a heat block set at 25°C. Finally, 90 µL of cell supernatants from each sample was transferred to new Eppendorf tubes and centrifuged at 20000 × g for 20 min at 4°C to pellet cell debris along with precipitated and aggregated proteins for western blot.

### *In vivo* tumor model

Female NOG mice (5-weeks-old) were obtained from Saeronbio Inc. (Uiwang-si, Gyeonggi-do, Republic of Korea) for glioma cell line injection and drug treatment. For tumor implantation, 5 × 10⁶ U-87MG cells were suspended in 100 μL of DPBS; and injected into the flank of each mouse using a 26G needle. Once the average tumor volume reached 60 mm³, tumor-bearing mice were randomly assigned to four groups: control, TMZ-treated, NNC 05-2090-treated, and TMZ + NNC 05-2090 combination-treated. TMZ was administered orally, and NNC 05-2090 was injected intraperitoneally every three days, for a total of eight injections. Tumor volume was measured twice weekly using a digital caliper and calculated using the formula: L × W × H × 0.5 (L = length, W = width, H = height; all in millimeters). All mice were euthanized five weeks after U-87MG cell implantation. All animal experiments were conducted in accordance with the approved procedures of the Korea Research Institute of Bioscience and Biotechnology (Approval Number: KRIBB-AEC-24100). All animal experiments were conducted in accordance with the NIH Guide for the Care and Use of Laboratory Animals, and all procedures adhered to the ARRIVE guidelines for the ethical use of animals in research.

### Bioinformatic analysis

Publicly available transcriptomic and clinical datasets were analyzed to investigate the expression patterns and clinical significance of NMUR2 and NMU in glioma. We utilized Gene Expression database of Normal and Tumor tissues (GENT2; http://gent2.appex.kr/gent2/) to compare NMUR2 expression between normal and brain tumor tissues. Additionally, RNA-sequencing-based transcriptomic profiles and corresponding clinical data from the cancer genome atlas (TCGA) low-grade glioma (LGG) and glioblastoma (GBM) cohort were retrieved from the UCSC Xena portal (http://xena.ucsc.edu).

### Combination index analysis

The interaction between NNC 05-2090 and temozolomide (TMZ) was evaluated using combination index (CI) analysis as previously described 32. CI values were calculated based on the doses of each drug used in combination relative to the doses of the individual drugs required to achieve the same level of growth inhibition. CI values were interpreted as follows: CI < 1, synergism; CI = 1, additive effect; and CI > 1, antagonism.

### Statistical analysis

Statistical analyses were performed using GraphPad Prism 10.4. All data are presented as the mean ± standard deviation (SD), unless otherwise indicated. The unpaired Student's t-test was used for analysis of differences between two groups. For comparison of multiple groups, one-way ANOVA or two-way ANOVA were performed depending on the experimental design, followed by multiple comparison tests, including Sidak's, Dunnett's, and Tukey's multiple comparisons tests. ^*^P < 0.05, ^**^P < 0.01, ^***^P < 0.001.

## Supplementary Material

Supplementary figures and table.

## Data Availability

The datasets used and/or analyzed in the current study are available from the corresponding author upon reasonable request.
