# Supplementary material for: Pharmacological Blockade of NMUR2 Suppresses Glioma Growth by Inhibiting STAT5-Mediated Transcription of Cell Cycle-associated Genes
Source: Int J Biol Sci. 2026 Jul 30;22(13):7151–72. doi: 10.7150/ijbs.135596 (PMC13540699; doi:10.7150/ijbs.135596)
Supplement: Supplementary file 1 [file ijbsv22p7151s1.pdf]

Supplementary Information

Pharmacological Blockade of NMUR2 Suppresses Glioma Growth by Inhibiting STAT5-Mediated Transcription of Cell Cycle-associated Genes

Yuna Roh, Taesang Son, Tae-Hee Han, Sarang Kim, Eunsun Jung, Jin-Seong Hwang, Joo-Young Im, Mi-Jung Kang, Mun Jeong Cho, Jung Hwa Lim, Cho-Rok Jung, Moo-Seung Lee, Hyun-Soo Cho, Dae-Soo Kim, Mi-Young Son, Seon-Young Kim, Seon-Kyu Kim*, Hyun Seung Ban*, Jang-Seong Kim* and Tae-Su Han*

***Corresponding authors:**

Tae-Su Han, Biotherapeutics Translational Research Center, Korea Research Institute of Bioscience and Biotechnology, 125 Gwahak-ro, Yuseong-gu, Daejeon 34141, Republic of Korea. Phone: +82-42-860-4297; E-mail: tshan@kribb.re.kr

Jang-Seong Kim, Biotherapeutics Translational Research Center, Korea Research Institute of Bioscience and Biotechnology, 125 Gwahak-ro, Yuseong-gu, Daejeon 34141, Republic of Korea. Phone: +82-42-860-4001; E-mail: jangskim@kribb.re.kr

Hyun Seung Ban, Biotherapeutics Translational Research Center, Korea Research Institute of Bioscience and Biotechnology, 125 Gwahak-ro, Yuseong-gu, Daejeon 34141, Republic of Korea. Phone: +82-42-879-8176; E-mail: banhs@kribb.re.kr

Seon-Kyu Kim, Aging Convergence Research Center, Korea Research Institute of Bioscience and Biotechnology, 125 Gwahak-ro, Yuseong-gu, Daejeon 34141, Republic of Korea. Phone: +82-42-879-8107; E-mail: seonkyu@kribb.re.kr

Supplementary Figures

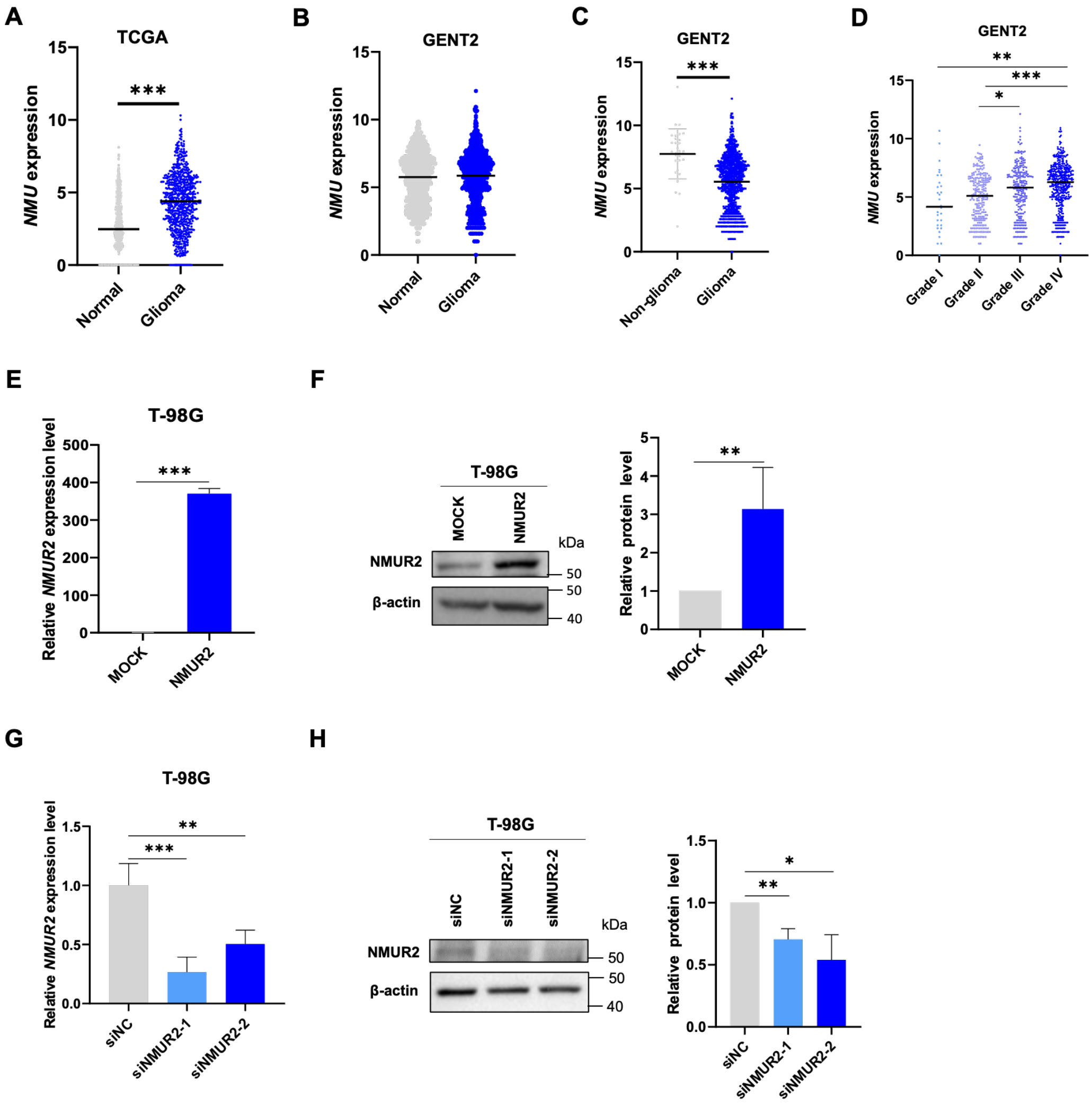

Figure S1. NMU expression in glioma datasets and NMUR2 expression in T-98G cells following overexpression or knockdown.

A. *NMU* expression in human normal brain tissues (n = 1141) and glioma tissues (LGG + GBM, n = 689) based on TCGA dataset analysis. B-D. *NMU* expression analysis using the GENT2 database. B. Comparison of *NMU* expression between normal brain tissues (n = 873) and glioma tissues (n = 946). C. Comparison of *NMU* expression between non-glioma brain tumor samples (n = 32) and glioma tissues (n = 946). D. *NMU* expression according to glioma grade. E-F. NMUR2 expression in T-98G cells transfected with empty vector or NMUR2 expression vector. E. *NMUR2* mRNA expression determined by qPCR. F. NMUR2 protein expression determined by western blotting. G-H. *NMUR2* expression in T-98G cells transfected with siNC or siNMUR2. G. *NMUR2* mRNA expression determined by qPCR. H. NMUR2 protein expression determined by western blotting. Data are presented as means ± SD (*$P < 0.05$, **$P < 0.01$, ***$P < 0.001$).

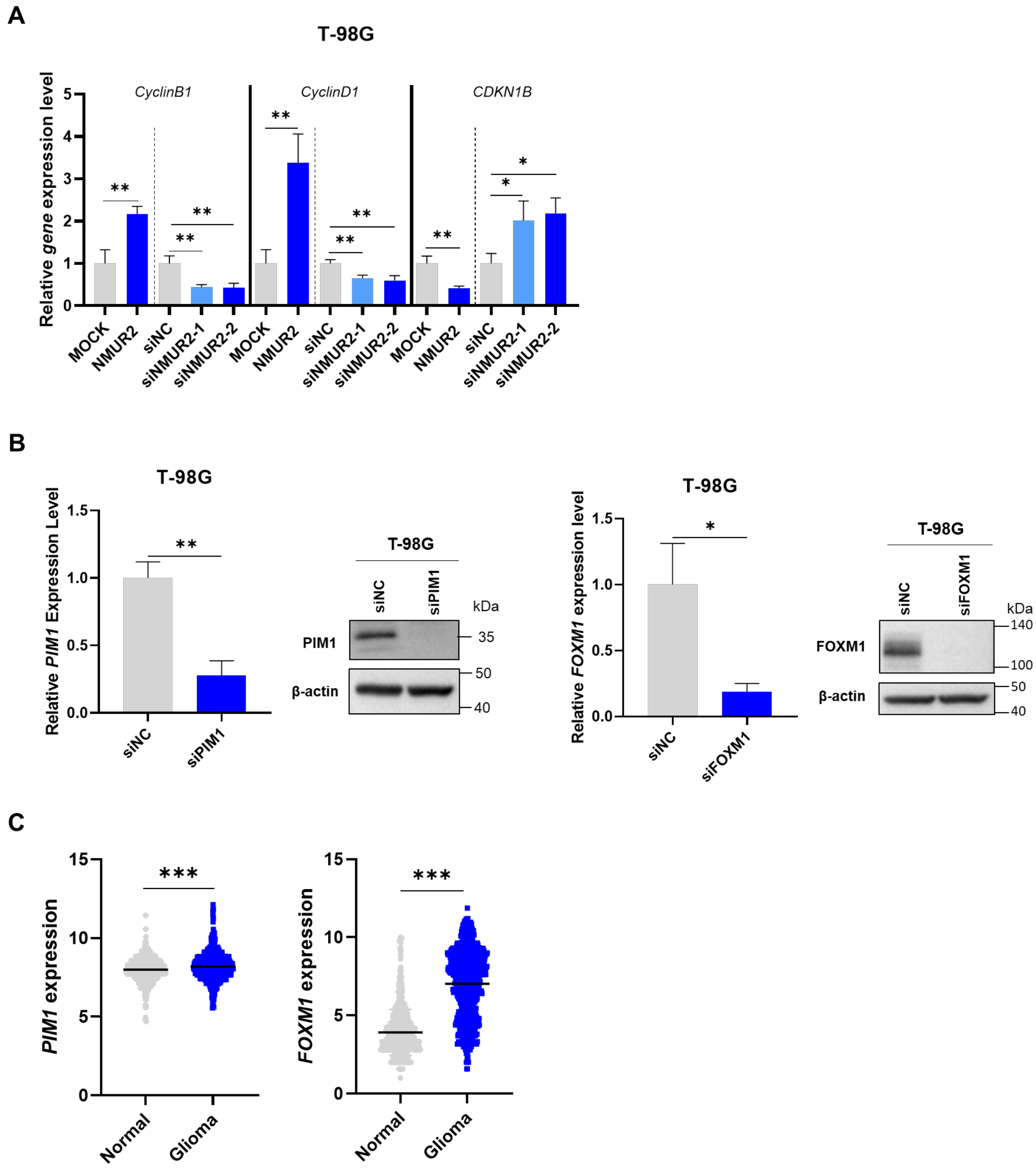

Figure S2. NMUR2 expression is involved in the regulation of the cell cycle.

A. The mRNA expression levels of *CyclinD1* and *CyclinB1* (cell cycle progression genes) and *CDKN1B* (a cell cycle repression gene) were analyzed using RT-qPCR in T-98G cells with NMUR2 overexpression or knockdown. B. PIM1 and FOXM1 expression in T-98G cells transfected with siNC, siPIM1, or siFOXM1. mRNA expression was determined by RT-qPCR, and protein expression was determined by western blotting. C. *PIM1* (left) and *FOXM1* (right) expression in human normal brain tissues (n = 873) and glioma tissues (n = 946) based on GENT2 database analysis (http://gent2.appex.kr/gent2/). Data are presented as means ± SD (*$P < 0.05$, **$P < 0.01$, ***$P < 0.001$).

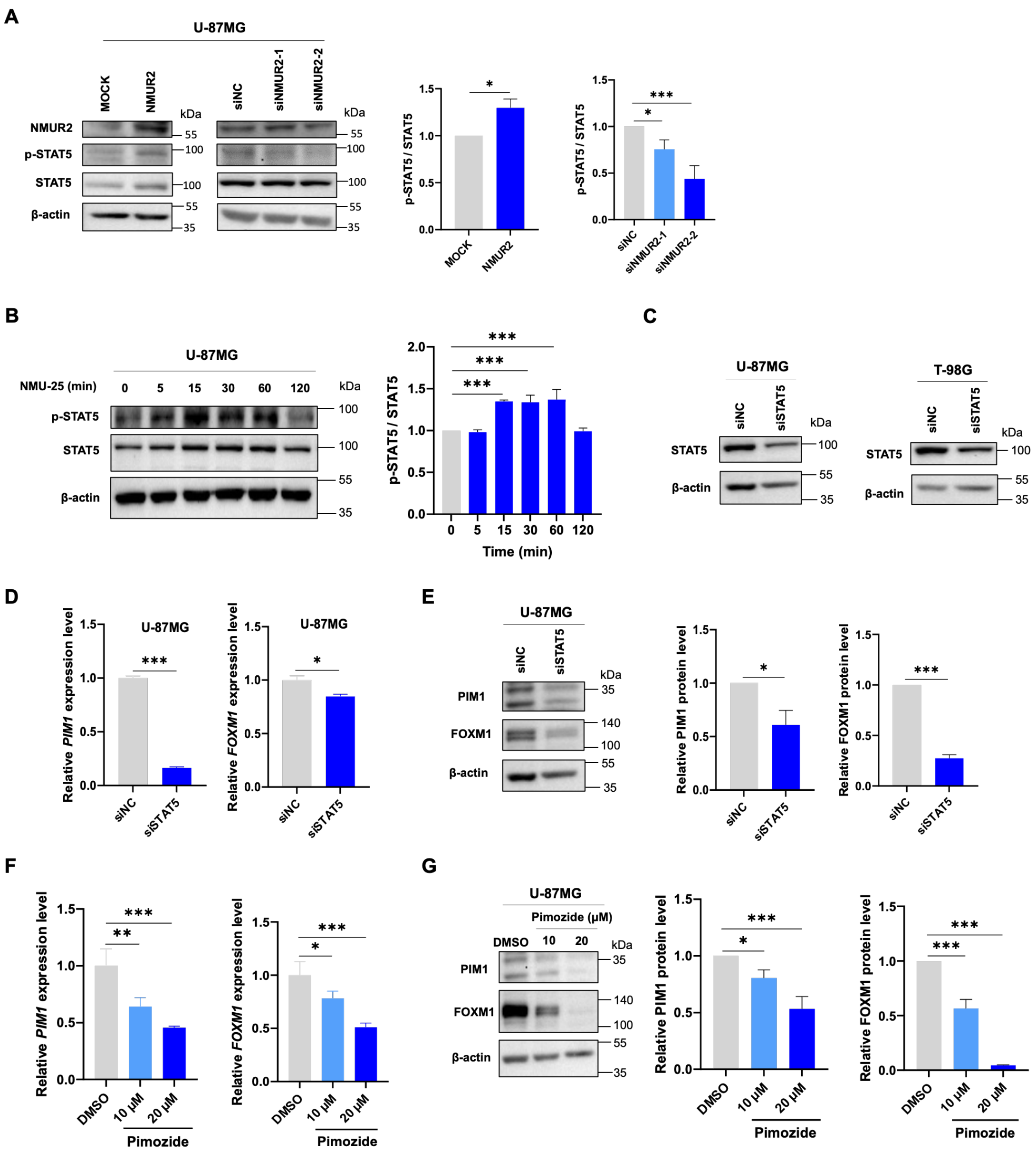

Figure S3. NMUR2-associated STAT5 activation and STAT5-dependent regulation of PIM1 and FOXM1 expression in glioma cells.

A. Phospho-STAT5 and total STAT5 levels in U-87MG cells following NMUR2 overexpressio n or knockdown, determined by western blotting. B. Time-dependent phosphorylation of STAT5 in U-87MG cells following NMU-25 treatment (0, 5, 15, 30, 60, and 120 min), determined by western blotting. Phospho-STAT5 levels were quantified relative to total STAT5. C. STAT5 protein expression in U-87MG and T-98G cells transfected with non-targeting control siRNA (siNC) or STAT5-targeting siRNA (siSTAT5), determined by western blotting. D-E. PIM1 and FOXM1 expression in U-87M G cells following siRNA-mediated STAT5 knockdown. D. *PIM1* and *FOXM1* mRNA expressi on determined by RT-qPCR. E. PIM1 and FOXM1 protein expression determined by western

blotting. F-G. PIM1 and FOXM1 expression in U-87MG cells treated with the indicated conc entrations of Pimozide. F. *PIM1* and *FOXM1* mRNA expression determined by RT-qPCR. G. PIM1 and FOXM1 protein expression determined by western blotting. Data are presented as means ± SD (*$P < 0.05$, **$P < 0.01$, ***$P < 0.001$).

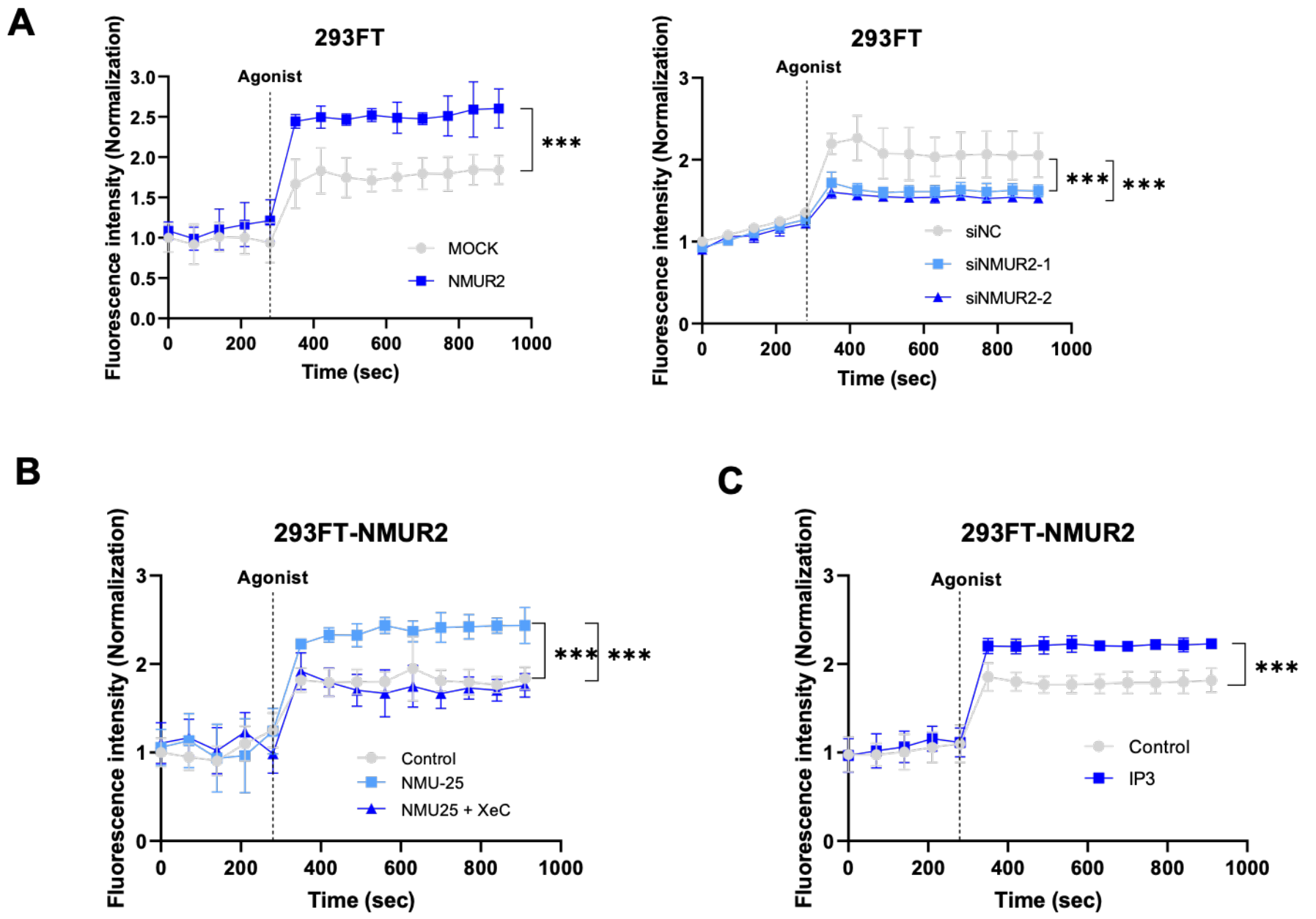

Figure S4. Intracellular Ca^{2+} analysis in 293FT cells following modulation of NMUR2 signaling.

A-C. Intracellular Ca^{2+} levels in 293FT cells measured using Green FLUOFORTE Dye. A. Intracellular Ca^{2+} levels in 293FT cells following NMUR2 overexpression or knockdown. B. Intracellular Ca^{2+} levels in 293FT cells treated with NMU-25 and Xestospongin C. C. Intracellular Ca^{2+} levels in 293FT cells treated with IP3. Data are presented as means ± SD (***$P < 0.001$).

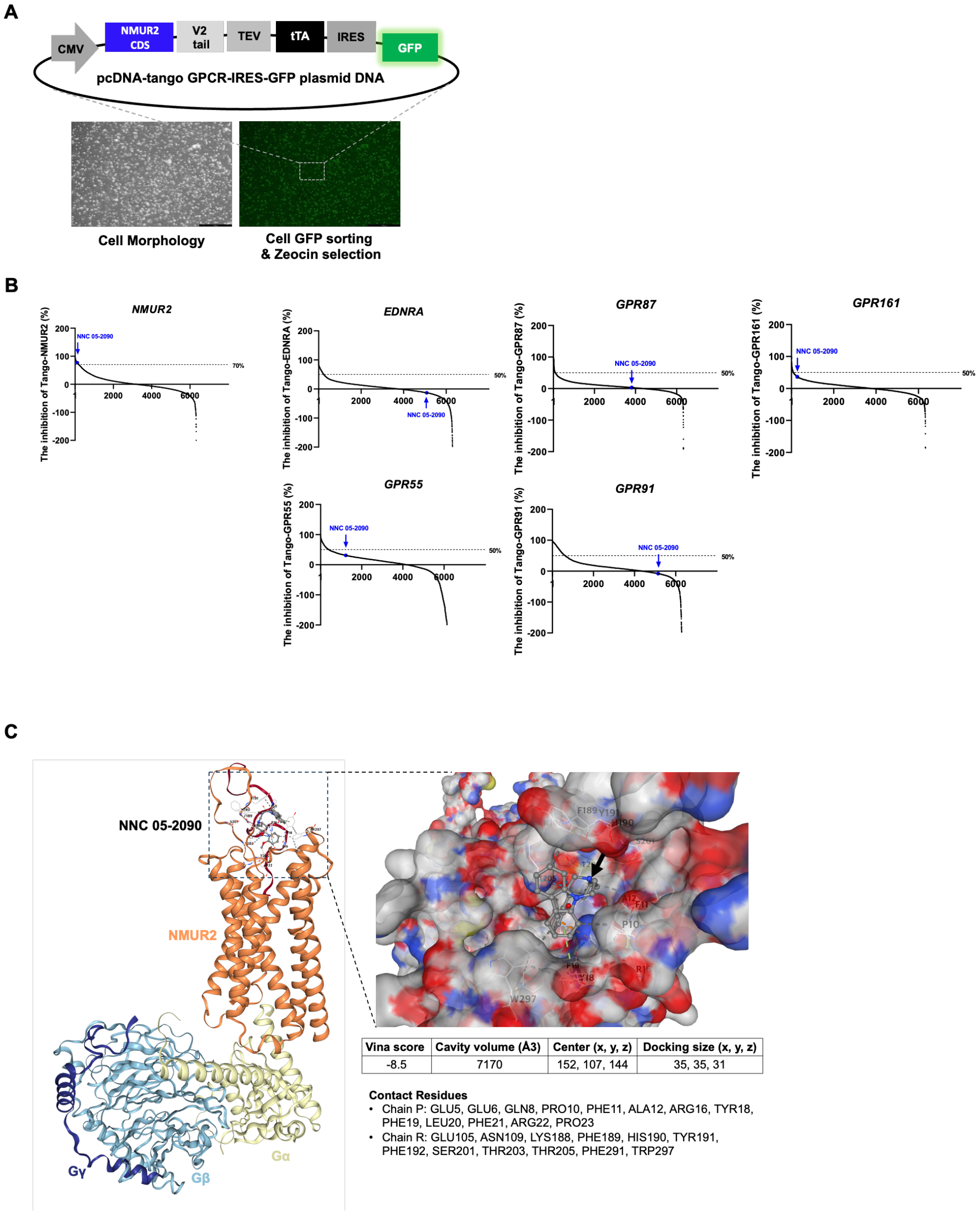

Figure S5. Construction of the NMUR2-specific PRESTO-TANGO screening system and evaluation of NNC 05-2090 selectivity.

A. Schematic representation of the pIRES-EGFP-NMUR2 plasmid and validation of the stable HTLA-NMUR2 cell line expressing GFP. The optimized NMUR2 TANGO coding sequence was inserted into the coding region of the pIRES-EGFP vector derived from the pcDNA3.1 backbone. B. Inhibition rates of NNC 05-2090 in NMUR2 and other GPCR assays. Luciferase inhibition rates were used to evaluate compound activity across receptors. C. Representative docking model of NNC 05-2090 in NMUR2 generated using CB-Dock2 with the NMUR2 structure (PDB ID: 7XK8). The predicted binding pocket is shown with the docked ligand, together with the corresponding vina score (-8.5 kcal/mol) and cavity volume (7170) obtained from CB-Dock2.

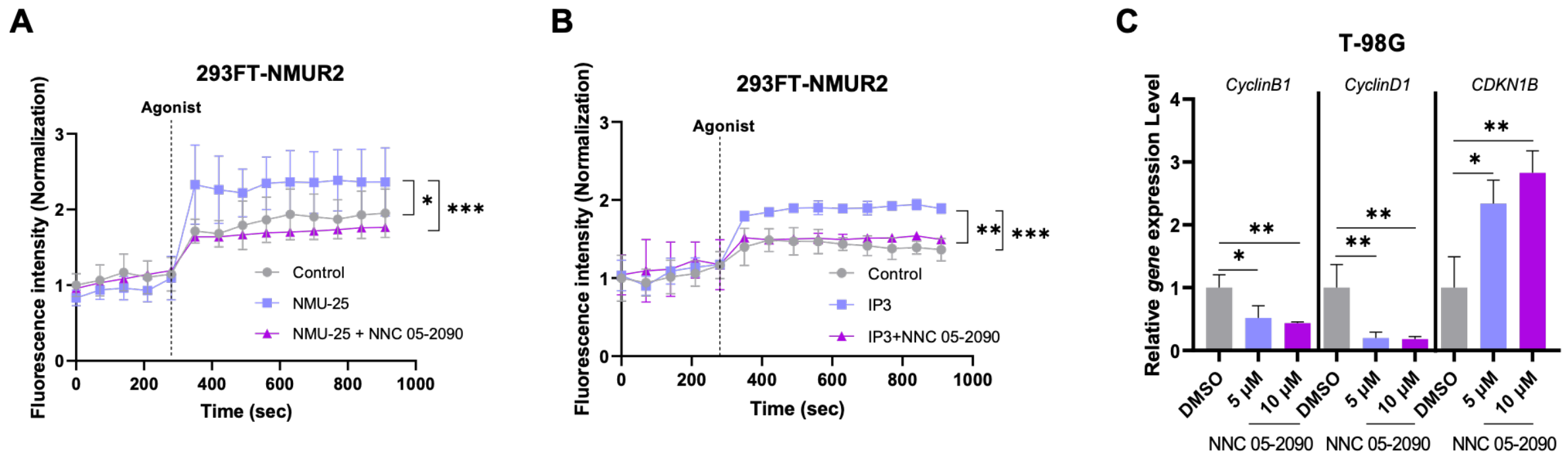

Figure S6. Intracellular Ca^{2+} analysis and cell cycle-associated gene expression following NNC 05-2090 treatment.

A-B. Intracellular Ca^{2+} levels in 293FT cells measured using Green FLUOFORTE Dye. A. Intracellular Ca^{2+} levels in cells treated with NMU-25 or co-treated with NMU-25 and NNC 05-2090. B. Intracellular Ca^{2+} levels in cells treated with IP3 or co-treated with IP3 and NNC 05-2090. C. *Cyclin B1*, *Cyclin D1*, and *CDKN1B* mRNA expression in T-98G cells treated with the indicated concentrations of NNC 05-2090, determined by RT-qPCR. Data are presented as means ± SD (*$P < 0.05$, **$P < 0.01$, ***$P < 0.001$).

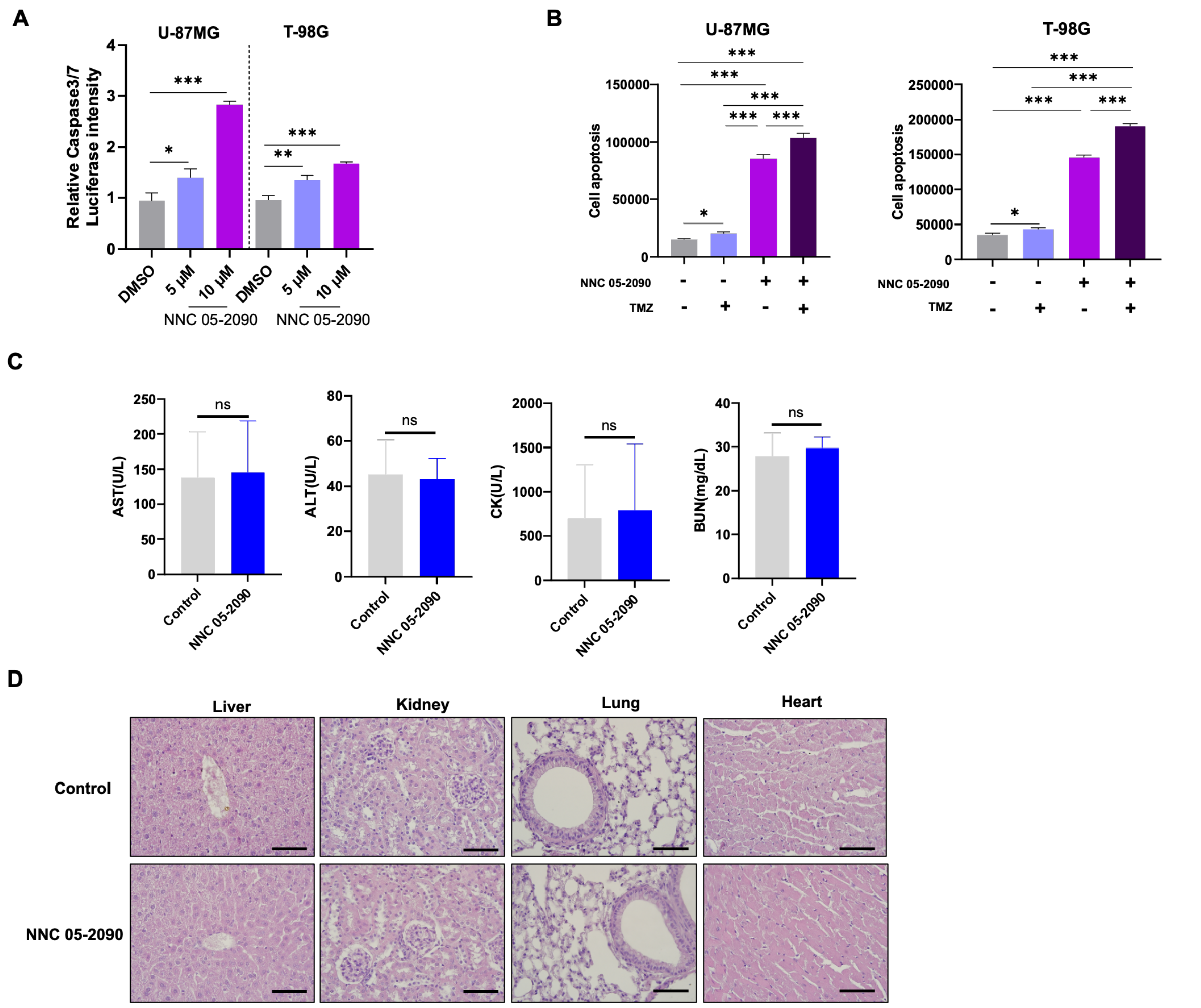

Figure S7. Cell apoptosis analysis in glioma cells and serum chemistry and histopathological analysis in control and NNC 05-2090-treated mice.

A. Cell apoptosis in glioma cell lines treated with the indicated concentrations of NNC 05-2090. Cell apoptosis was assessed by Caspase-Glo 3/7 assay. B. Cell apoptosis in U-87MG (left) and T-98G (right) cells treated with NNC 05-2090 (10 μM), temozolomide (TMZ, 1 mM), or the combination of NNC 05-2090 and TMZ. Cell apoptosis was assessed by Caspase-Glo 3/7 assay. C. Serum levels of AST, ALT, CK, and BUN in control and NNC 05-2090-treated mice. Data are presented as mean ± SD. D. Representative H&E-stained sections of liver, kidney, lung, and heart from control and NNC 05-2090-treated mice. Scale bar, 50 μm. AST, aspartate transaminase; ALT, alanine transaminase; CK, creatine kinase; BUN, blood urea nitrogen. Data are presented as means ± SD (*$P < 0.05$, **$P < 0.01$, ***$P < 0.001$).

Supplementary Table S1. List of sequences

	Gene	Primer (Sense)	Primer (antisense)
**NMUR2 CDS primers**	NMUR2	TTTGAATTCGCCACCATGTCA<br>GGGATGGAA	GTTCCGCGGTCAGGTTTTGTT<br>AAGTGGAA
**siRNA sequences**	Negative control siRNA (siNC)	UUCUCCGAACGUGUCACGUT<br>T	ACGUGACACGUUCGGAGAAT<br>T
	siNMUR2-1	AGAAUGCUUCCUGGAUCUA	UAGAUCCAGGAAGCAUUCU
	siNMUR2-2	GCUGACCGAAGAUAUAGGU	ACCUAUAUCUUCGGUCAGC
**qPCR primers**	GAPDH	CTCTGCTCCTCCTGTTCGAC	TTAAAAGCAGCCCTGGTGAC
	NMUR2	AGATGTGGCGCAACTACCC	CGAAGCACACGGTCTCAAAG<br>A
	PIM1	GAGAAGGACCGGATTTCCGA<br>C	CAGTCCAGGAGCCTAATGACG
	FOXM1	ATACGTGGATTGAGGACCACT	TCCAATGTCAAGTAGCGGTTG
	Cyclin D1	GCATGTTCGTGGCCTCTAAG	CGTGTTTGCGGATGATCTGT
	Cyclin B1	TGAGGAAGAGCAAGCAGTCA	CCAAATGCGTGTCCTCAGAG
	CDKN 1B	GCAAGTACGAGTGGCAAGAG	CCAAATGCGTGTCCTCAGAG
**Promoter assay**	PIM1	GGCCGGTACCTGAGCTCGCTA<br>GCGTGAAAACTGGTTAAATCA<br>AGCGCAC	CGAGGCCAGATCTTGATATCC<br>TCGAGGGAGTGGAGGAGTCAG<br>GCCTGGAACAGAAC
	FOXM1	GGCCGGTACCTAGCTCGCTAG<br>CGTCTGTGCCCCTCTTCCAGGA<br>TTGGGCTG	CGAGGCCAGATCTTGATATCC<br>TCGAGGAACGATCGTCTGCTCC<br>CCCCGATTAG
**NMUR2 Tango sequences**	NMUR2 optimized sequences	ATGTCCGGGATGGAAAAACTTCAGAACGCCAGTTGGATTTACCAA<br>CAGAAGCTGGAGGATCCTTTCCAGAAGCACCTGAACTCTACAGAG<br>GAATACCTGGCTTTCCTCTGCGGCCCAAGACGATCCCACTTCTTTT<br>TGCCCGTGTCAGTCGTGTACGTGCCCATTTTTGTGGTCGGCGTCAT<br>AGGGAACGTGCTTGTGTGCCTGGTTATTCTGCAGCATCAGGCCAT<br>GAAAACTCCAACAAATTATTACCTCTTTAGTCTGGCAGTGTCTGAT<br>CTGCTCGTGCTTCTGTTGGGCATGCCTCTGGAGGTATACGAGATGT<br>GGAGAAATTATCCTTTTCTGTTTGGCCCCGTGGGTTGTTACTTCAA<br>GACTGCTCTTTTTGAGACCGTCTGTTTCGCTTCCATCCTCAGCATA<br>ACGACAGTGTCTGTGGAACGCTATGTCGCCATCCTGCACCCATTTC<br>GAGCTAAGCTGCAATCCACCCGCCGGCGGGCCCTCAGAATTCTGG<br>GCATCGTGTGGGGCTTCTCAGTTCTGTTTTCCCTGCCAAATACTAG<br>TATTCATGGAATTAAATTTCATTACTTTCCTAATGGGTCCCTTGTGC<br>CCGGCTCTGCCACATGCACCGTTATTAAGCCTATGTGGATCTACAA<br>CTTCATTATCCAAGTCACAAGTTTTTTGTTCTACCTGTTGCCGATGA<br>CAGTTATATCAGTACTGTACTACCTCATGGCCCTGCGGCTGAAAAA<br>AGACAAAAGCCTTGAGGCAGATGAGGGGAATGCCAACATCCAGC<br>GCCCTTGTCGCAAATCTGTGAACAAGATGCTTTTTGTGTTGGTACT<br>GGTTTTTGCTATTTGCTGGGCCCCATTCCACATTGACCGGCTGTTTT<br>TTTCTTTTGTCGAGGAATGGTCTGAAAGCCTTGCTGCCGTGTTCAA<br>CCTGGTGCACGTCGTTTCAGGCGTATTCTTTTATCTGTCCTCCGCC<br>GTTAATCCTATAATTTACAATCTGCTTAGCAGAAGATTCCAGGCTGC<br>ATTCCAGAATGTAATCTCATCATTTCACAAACAGTGGCATAGTCAG<br>CACGACCCCCAGCTCCCACCGGCTCAGAGAAATATCTTTCTCACG<br>GAGTGCCACTTCGTGGAACTCACCGAAGATATCGGGCCGCAGTTT<br>CCCTGTCAGTCTTCAATGCACAATAGCCACCTGCCTGCCGCCCTGA<br>GTAGCGAGCAGATGTCCCGGACTAACTATCAGTCCTTCCACTTCAA<br>TAAGACG	
